# Efficient training of spiking neural networks with temporally-truncated local backpropagation through time

**DOI:** 10.3389/fnins.2023.1047008

**Published:** 2023-04-06

**Authors:** Wenzhe Guo, Mohammed E. Fouda, Ahmed M. Eltawil, Khaled Nabil Salama

**Affiliations:** ^1^Sensors Lab, Advanced Membranes and Porous Materials Center (AMPMC), Computer, Electrical and Mathematical Sciences and Engineering (CEMSE) Division, King Abdullah University of Science and Technology, Thuwal, Saudi Arabia; ^2^Communication and Computing Systems Lab, Computer, Electrical and Mathematical Sciences and Engineering (CEMSE) Division, King Abdullah University of Science and Technology, Thuwal, Saudi Arabia; ^3^Center for Embedded & Cyber-Physical Systems, University of California, Irvine, Irvine, CA, United States

**Keywords:** backpropagation through time, deep learning, energy-efficient training, local learning, neuromorphic computing, spiking neural networks

## Abstract

Directly training spiking neural networks (SNNs) has remained challenging due to complex neural dynamics and intrinsic non-differentiability in firing functions. The well-known backpropagation through time (BPTT) algorithm proposed to train SNNs suffers from large memory footprint and prohibits backward and update unlocking, making it impossible to exploit the potential of locally-supervised training methods. This work proposes an efficient and direct training algorithm for SNNs that integrates a locally-supervised training method with a temporally-truncated BPTT algorithm. The proposed algorithm explores both temporal and spatial locality in BPTT and contributes to significant reduction in computational cost including GPU memory utilization, main memory access and arithmetic operations. We thoroughly explore the design space concerning temporal truncation length and local training block size and benchmark their impact on classification accuracy of different networks running different types of tasks. The results reveal that temporal truncation has a negative effect on the accuracy of classifying frame-based datasets, but leads to improvement in accuracy on event-based datasets. In spite of resulting information loss, local training is capable of alleviating overfitting. The combined effect of temporal truncation and local training can lead to the slowdown of accuracy drop and even improvement in accuracy. In addition, training deep SNNs' models such as AlexNet classifying CIFAR10-DVS dataset leads to 7.26% increase in accuracy, 89.94% reduction in GPU memory, 10.79% reduction in memory access, and 99.64% reduction in MAC operations compared to the standard end-to-end BPTT. Thus, the proposed method has shown high potential to enable fast and energy-efficient on-chip training for real-time learning at the edge.

## 1. Introduction

In recent years, deep learning surged as a method for solving various complex tasks, such as visual processing (Li Z. et al., 2021), language processing (Young et al., 2018), object detection (Zhao et al., 2019), and medical diagnostics (Mahmud et al., 2018), making it the most promising and dominant approach. The remarkable performance of deep learning comes at the expense of substantial energy consumption resulting from intensive full-precision matrix multiply-accumulate (MAC) operations in artificial neural networks (ANNs). This drawback holds back deep learning algorithms from being deployed in resource-constrained platforms, such as edge devices. Inspired by the biological nervous system, spiking neural networks (SNNs) have attracted ever-growing attention from research communities for their superior energy efficiency to ANNs. Information in SNNs is transmitted and processed on the occurrence of a spike or an event. The large spike sparsity and simple synaptic operations in SNNs give rise to low energy consumption. SNNs have been explored in a broad range of applications, such as pattern recognition (Guo et al., 2021; Yu et al., 2021), object detection (Kim et al., 2020), navigation (Beyeler et al., 2015), and motor control (Naveros et al., 2020). Based on SNNs, neuromorphic computing systems have been proposed as an alternative computing paradigm to the traditional Von Neumann systems (Davies et al., 2018; Abderrahmane et al., 2020; Höppner et al., 2021).

**Figure 1:**
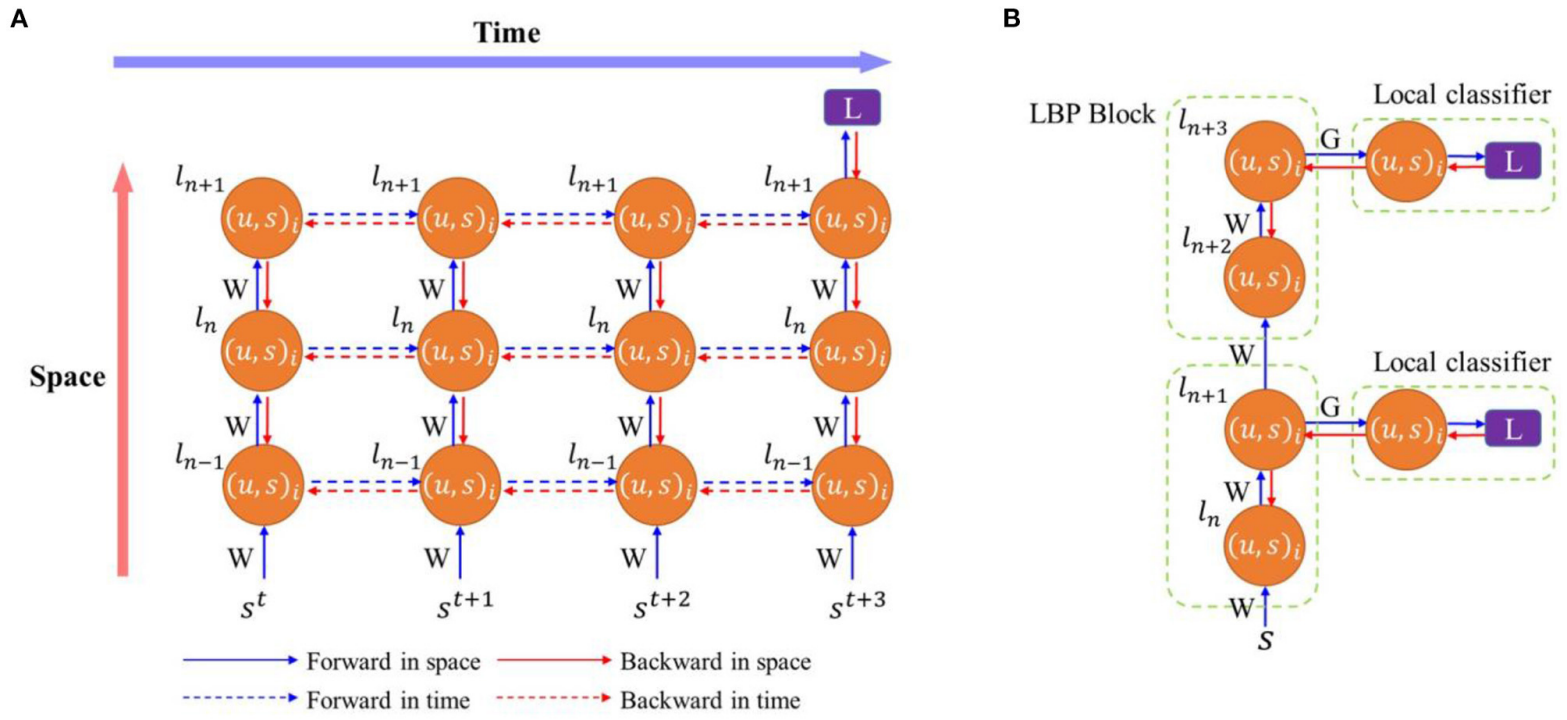
**(A)** Training SNNs with backpropagation through time. Training processes are unfolded in time. **(B)** Training SNNs with local backpropagation. Each node represents a spiking neuron layer where *l* is the layer index, *i* is the neuron index, *u* is the membrane potential, and *s* is the spike. *W* is weights between layers in the main network and *G* is the weights between a network layer and a local classifier. *L* is a loss function.

Training SNNs has been a significant challenge in exploiting the full potential of SNNs due to complex neural dynamics and discontinuous spikes (Tavanaei et al., 2019). The lack of efficient and effective training algorithm limits the use of SNNs in complex real-world tasks. Existing training algorithms can be categorized into two general approaches: indirect training and direct training. The indirect training relies on the conversion from a well-trained ANN model to an architecturally equivalent SNN model. The learned parameters in the DNN are directly transferred to the SNN, while the activations in the DNN corresponds to the firing rates of SNN neurons (Diehl et al., 2015; Sengupta et al., 2019; Wu et al., 2021). The conversion-based method generally requires a high inference latency to reach comparable accuracy to the equivalent ANNs. Although recent efforts managed to reduce the inference latency by tens to hundreds of times, the inference is still slower than direct training methods (Ding et al., 2021; Liu et al., 2022; Meng et al., 2022). Since the conversion is based on ANNs, the converted SNNs cannot directly process neuromorphic data. Moreover, only the inference phase is performed in SNNs, so this method is not able to effectively exploit the rich temporal dynamics of SNNs and provides little insight into the underlying training mechanism of biological brains. Direct training methods can be categorized into unsupervised and supervised approaches. The unsupervised training methods, such as spike-timing-dependent plasticity (STDP), are inspired by the biological nervous systems, modifying weights in terms of local synaptic activities (Bi and Poo, 1998). Without supervision signals, these methods exhibit inferior performance (Diehl and Cook, 2015; Kheradpisheh et al., 2018; Srinivasan et al., 2018). The supervised training methods are mainly based on gradient descent optimization, such as SpikeProp (Bohté et al., 2000) and Tempotron (Gütig and Sompolinsky, 2006). STDP was also incorporated in gradient-descent-based methods for different purposes, such as pre-training (Lee et al., 2018), fine tuning (Furuya and Ohkubo, 2021), and efficient local weight updates (Tavanaei and Maida, 2019; Liu et al., 2021). Different supervised training mechanisms were derived from different neural coding schemes, such as rate coding and time-to-first-spike coding. The temporal-coding based methods consider the exact firing time of the first spike as the essential information and compute a loss between the exact time and the desired time (Kheradpisheh and Masquelier, 2020; Mirsadeghi et al., 2021; Park and Yoon, 2021). Whereas, rate-coding based training performs optimization based on firing rates. One type of such methods derives a transfer function that formularizes the accumulated effect of spikes, like firing activity or rate, from the event-based update of membrane potential (Lee et al., 2016, 2020; Jin et al., 2018). Due to the similarity between SNNs and recurrent neural networks (RNNs), it is not surprising that the training algorithm, backpropagation through time (BPTT), used in RNNs can be borrowed for SNNs (He et al., 2020). The training process is depicted in Figure 1A. During the forward process, neural states in SNNs are iteratively updated with both spatial and temporal inputs throughout the whole time window. The backward process starts at the end of the training window when the loss function is computed. BPTT has been demonstrated to be very effective in training SNNs by considering the spatio-temporal dynamics (Shrestha and Orchard, 2018; Wu et al., 2018, 2019; Deng et al., 2020; Kim and Panda, 2021; Zheng et al., 2021). SNNs trained with BPTT closely approach ANNs in classification performance on various datasets. More importantly, BPTT allows SNNs to be scaled to very deep networks (50 layers) and hence empowers SNNs to solve more complex tasks. Compared with the conversion-based methods, direct training methods can also achieve comparable accuracy to ANNs on various frame-based datasets and even better accuracy on neuromorphic datasets. The simulation latency can be effectively reduced to a few time steps while competitive accuracy is retained. Direct training can be applied under different neural coding methods in SNNs, such as time-to-first-spike coding, to realize very efficient training and inference. Additionally, it provides a way for real-time on-chip learning at the edge.

Typically, in the standard BP algorithm, errors are propagated backward in a layer-by-layer fashion to update training parameters. The activation values need to be saved during the forward pass and read out for parameter updates during the backward pass. Despite the effectiveness of the standard BP algorithm, the network suffers from frequent memory access, computational inefficiency, and long training time. Thus, various local BP (LBP) algorithms were proposed to tackle the aforementioned issues (Marquez et al., 2018; Mostafa et al., 2018; Nøkland and Eidnes, 2019; Wang et al., 2021). An example of the block-wise LBP training is illustrated in Figure 1B. The LBP algorithms attach a classifier to a block of layers (or a single layer) in the network and train each block separately and simultaneously. Since training happens locally, intermediate states can be saved in buffers temporally before parameter updates, eliminating the need for memory storage and access (Mostafa et al., 2018). Moreover, LBP divides the whole network into gradient-isolated modules, making the hardware design scalable because the network can be built by cascading the same local training module. However, LBP suffers from inferior performance compared to the standard BP because of information loss (Wang et al., 2021). Few works have considered LBP in SNNs. Kaiser et al. demonstrated the effectiveness of LBP in SNNs (Kaiser et al., 2020). In his work, a classifier with random weights was attached to each layer. The networks were trained by approximate BP with a surrogate gradient for the firing function at each time step. Temporal dependency in the backward update was completely ignored because of intractable gradient computation in the algorithm. Although competitive classification performance was achieved on one dataset against the state-of-the-art works, the method required to learn each input image in a very long time window, 500 time steps. This work failed to provide a fair comparison against the BPTT algorithm and generalize the effectiveness of the proposed training algorithm to deeper networks and complex datasets. Ma et al. experimented layerwise local training in spike-based BPTT to train SNNs (Ma et al., 2021). Good performance was achieved in various tasks but at the expense of high computational cost. Different from the discussed LBP, other forms of local training were also reported in SNNs (Kheradpisheh et al., 2018; Srinivasan et al., 2018; Tavanaei and Maida, 2019; Liu et al., 2021; Mirsadeghi et al., 2021). STDP performs weight updates based on the correlation between presynaptic spikes and postsynaptic spikes without the need for global signals. It is a simple and hardware-friendly training algorithm. In Kheradpisheh et al. (2018) and Srinivasan et al. (2018), the unsupervised STDP learning rule was applied in convolutional networks and achieved good accuracy on simple tasks, such as MNIST. However, it was limited to shallow networks. The work in Mirsadeghi et al. (2021) proposed a gradient descent algorithm that utilized the time-to-first-spike coding and trained neurons at each layer to fire at desired times. Weights were updated by using a local gradient descent formula by computing a local loss function between the actual firing times and the desired firing times. However, the desired firing times at each layer needed to be computed through a global error backpropagation process. Thus, overall, this method cannot achieve fully local updates. In contrast, the LBP used in our work utilized rate coding and trained the neurons at each layer to reach desired firing rates. The loss function was computed between local predictions and labels. There was no error backpropagation between layers. Following the same temporal-coding-based training algorithm, Liu et al. (2021) applied the STDP local learning mechanism to approximate the gradients of firing times with respect to the weights, i.e., ∂*t*^*l*^/∂*w*^*l*^, and the BP mechanism for error propagation. This training algorithm benefited from the STDP for local feature extraction and efficient weight updates. With global feedback enabled by the BP process, it achieved comparable accuracy to the state-of-the-art methods on different datasets. However, the complete gradients still relied on the errors backpropagated from the subsequent layers. It cannot solve the aforementioned issues of the end-to-end BP. Tavanaei and Maida (2019) proposed a temporally local and efficient STDP-based training algorithm. By comparing IF neurons to ReLU neurons, this method applied the BP mechanism to enable gradient descent optimization. It also incorporated STDP and anti-STDP learning mechanisms for weight update computation. However, in the spatial dimension, it still followed the end-to-end BP process to propagate the errors. Since the LBP method utilizes only local information without any global feedback and has been proven to be efficient and scalable in training, this work conducts detailed investigations into the performance of the LBP with the BPTT algorithm.

The BPTT algorithm dictates that the backward pass can only happen after the network moves forward throughout the whole time window. It requires the network to store the time evolutions of neural states, as the backward pass needs them to compute gradients at each step, which incurs a substantial memory footprint. The accumulation of gradients in a long time window can cause gradient exploding issues (Pascanu et al., 2013). When the local training method is applied together with BPTT in SNNs, the backward pass does not need the spatial gradients to be backpropagated from the next block, but it needs the temporal gradients to be backpropagated in time. So the backward pass has to wait for the forward pass to finish. Except for the last time step, the intermediate states cannot be saved in the buffer on chip, because it would not be used immediately. They have to be saved in the external memory and accessed during the backward pass. As a result, LBP loses its advantage over standard BP. Inspired from the idea in truncated BPTT (TBPTT) applied in RNNs (Williams and Zipser, 1995; Sutskever, 2013), we introduce temporal truncation in spiking BPTT to resolve the incompatibility issue between LBP and BPTT for training SNNs. TBPTT divides the training time window into many temporal chunks and runs BPTT for each chunk. It breaks the temporal restriction imposed on the backward pass, allowing for the advantages of LBP to be considerable. The smaller the chunk, the more significant contribution LBP can make. Additionally, temporal truncation is able to cut short computational graphs built for backward updates proportionally, leading to significant reduction in memory footprint.

In this work, we propose an efficient training method for SNNs by integrating local training methods with BPTT by introducing temporal truncation. The proposed method can significantly reduce memory footprint and access, and arithmetic operations with negligible performance loss. The training process can benefit from the proposed method both temporally and spatially. However, both LBP and TBPTT could suffer from inferior performance depending on the size of truncated chunks and the length of local blocks. Thus, we will investigate the impact of temporal truncation and spatial locality applied in BPTT on classification performance and computational cost in SNNs. With the proposed method, we aim to resolve the challenge that an effective on-chip training algorithm for SNNs is still not available for real-time applications. Our motivation is based on the following facts. BPTT is a promising training approach that can empower SNNs to be competitive with ANNs in large-scale implementations. Local BP-based training methods show great advantages of significantly reducing computational costs while retaining good algorithmic performance and scalability. They can potentially reduce the hardware complexity of the BPTT algorithm without incurring significant performance degradation, which provides a chance for us to develop an effective and efficient online-learning solution for SNNs that could be practically deployable. This is very meaningful for resource-constrained or latency-sensitive or power-limited computing platforms, such as edge devices and autonomous driving vehicles.

The main contributions are summarized as follows.

1) We introduce temporal truncation in BPTT to resolve the incompatibility issue between LBP and BPTT, and thus propose an efficient training algorithm for SNNs with significantly reduced memory footprint and access, and arithmetic operations.
2) We thoroughly explore the design space regarding the temporal truncation length and local training size and analyze their impact on classification performance and computational cost of different SNNs for various datasets.
3) We provide analytical models for predicting and estimating memory footprint and access, arithmetic operations on different hardware platforms.
4) We compare trainable classifiers and random classifiers applied in LBP and demonstrate that random classifiers do not provide considerable advantages while suffering from severe performance drop.

The rest of this article is organized as follows. Section 2 introduces neural models and the proposed training algorithm. Section 3 describes the details of experiments and presents classification results. Section 4 analyzes computational cost of the proposed algorithm and presents corresponding results. Section 5 summarizes our work and discusses limitations and future perspectives.

## 2. Methods

### 2.1. Neural models

Leaky integrate-and-fire neuron (LIF) model is widely used to model spiking neurons because it can accurately capture neural dynamics and has excellent computational efficiency. It consists of a linear equation and a threshold condition. The model can be written as


(1) $$\begin{array}{l} u_{i}^{t+1,n}=\tau u_{i}^{t,n}+\underset{j}{\sum }W_{ij}^{n}s_{j}^{t+1,n-1}-\theta s_{j}^{t,n} \end{array}$$

(2) $$\begin{array}{l} s_{i}^{t+1,n}=\text{Θ}\left(u_{i}^{t+1,n}-u_{th}\right) \end{array}$$

where $u_{i}^{t,n}$ and $s_{i}^{t,n}$ are the membrane potential and output spike of the neuron *i* in the layer *n* at time *t*, respectively, $W_{ij}^{n}$ is the synaptic weight between the neuron *i* in the layer *n* and the neuron *j* in the layer *n* − 1, τ is the leaky time constant, *u*_*th*_ is the threshold potential, θ is the reset constant, and Θ (·) is a unit step function. A soft reset mechanism is used to reset membrane potential once an output spike is generated.

### 2.2. Temporally-truncated local BPTT

**Figure 2:**
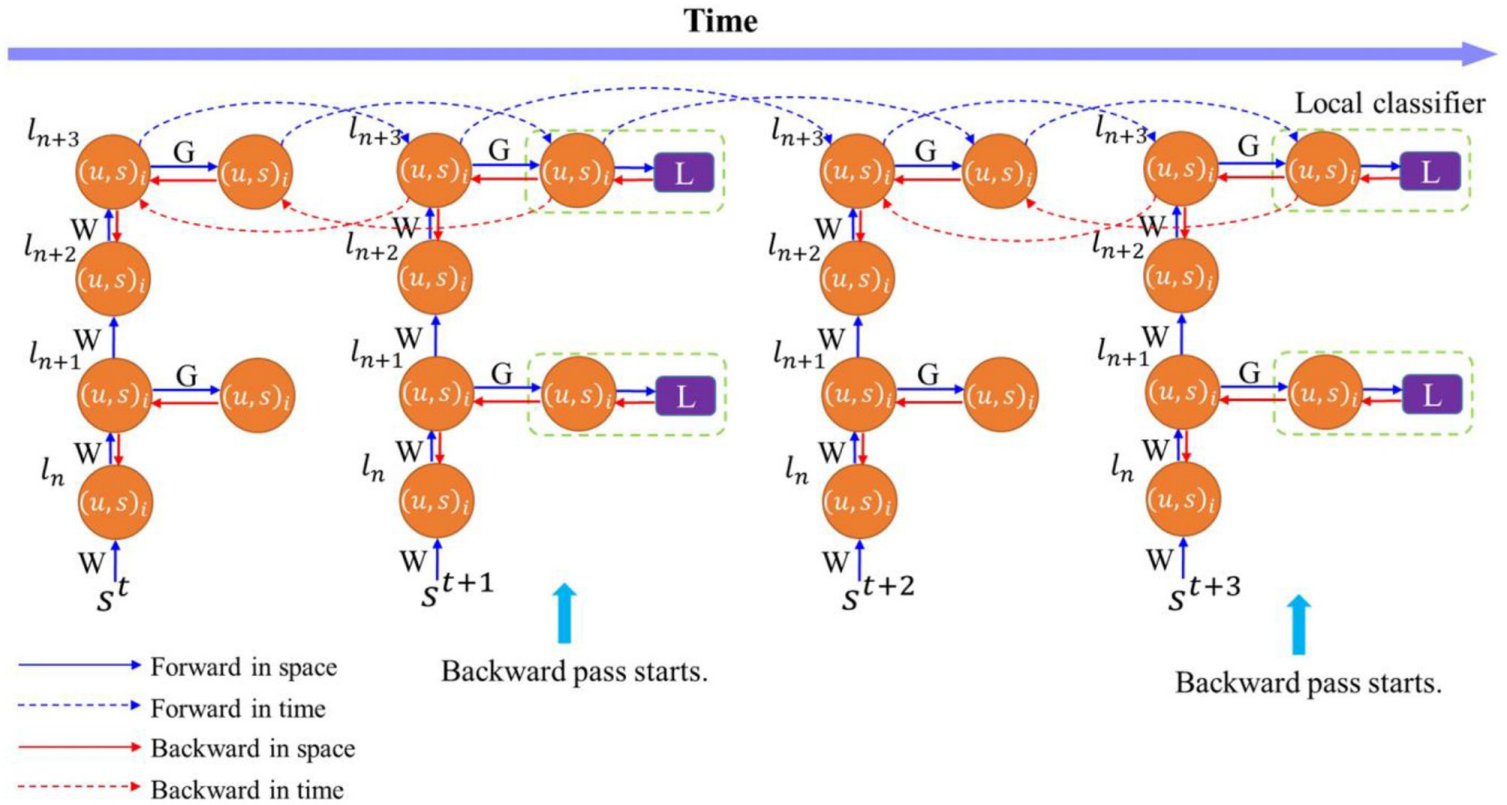
Training process of an SNN with temporally-truncated local BPTT unfolded in time. In this illustration, the length of the truncation interval and local block are both 2. The forward and backward update flows are indicated by the blue and red arrows, respectively. The update flows in time (dashed arrows) are only depicted for the top layer for clarity. W represents the weights between layers in the main network, and G is the weights between local blocks and classifiers.

We introduce temporal truncation in the BPTT algorithm together with local classifiers to jointly train SNNs. Figure 2 illustrates the proposed training method, where temporal truncation with a step size of 2 and two-layer local blocks are applied in BPTT. During forward pass, neuron states of the main network and local classifiers are updated iteratively in space and time, as indicated by blue arrows. Backward pass happens after every truncation interval. A loss is computed at each local classifier during the backward pass, and errors are propagated backward from classifiers spatially to local blocks and temporally to their previous states, as indicated by the red arrows. Inside a local block, errors are propagated in the same fashion. But the error flow stops between blocks, removing the backward dependency between blocks. This way eliminates the need to store intermediate states of the current block in external memory and makes it possible to execute forward pass and backward pass in parallel. The error flow is also discontinued between truncation intervals, which eliminates the need to store all the neural states updated in the previous intervals. The training process benefits from both temporal truncation and local learning in reducing the computational cost. However, both methods could also affect network performance, since temporal truncation removes the temporal dependency between truncated intervals during the backward pass and local training could potentially cause information loss. Accordingly, we define a variable pair (*k, n*), as the length of a temporal interval and the number of layers in a local block, respectively. We explore the design space of these two factors and analyze the impact on network performance.

Moreover, in local training methods, applying trainable local classifiers can retain high performance but add additional weight parameters to be trained, incurring training overhead. Using random weights in local classifiers was proposed to reduce the overhead but proven to be less effective in training networks (Mostafa et al., 2018). Thus, we provide a detailed analysis of the effect of trainable and random local classifiers in our proposed training algorithm.

Following the theoretic framework in Wu et al. (2018), we derive the essential equations used in the training algorithm as follows. Firstly, we define the loss function as the mean squared error between the time average firing rates of classifier neurons and target firing rates, expressed by


(3) $$\begin{array}{l} L=\frac{1}{N_{c}}\underset{i}{\sum }{\left(y_{i}-\frac{1}{k}\underset{t}{\sum }s_{c,\;i}\left(t\right)\right)}^{2} \end{array}$$

where *y*_*i*_ and *s*_*c,i*_(*t*) is the target firing rate of classifier neuron *i* and the actual firing rate at time *t*, respectively, and *N*_*c*_ is the number of classes. The average firing rate is calculated over each truncation interval. In this work, the target firing rate vector for the classifier is determined as a one-hot vector based on the target class. Then, we define the spike error, $\delta_{i}^{t,n}=\frac{\partial L}{\partial s_{i}^{t,n}}$, and the potential error, $\gamma_{i}^{t,n}=\frac{\partial L}{\partial u_{i}^{t,n}}$. Based on the two errors, the iterative backward update equations are given as,


(4) $$\begin{array}{l} \delta_{i}^{t,n}=\underset{m}{\sum }\gamma_{m}^{t,n+1}W_{mi}^{n}+\gamma_{i}^{t+1,n}\left(-\theta \right) \end{array}$$

(5) $$\begin{array}{l} \gamma_{i}^{t,n}=\delta_{i}^{t,n}{\text{Θ}}^{{}^{\prime }}\left(u_{i}^{t,n}-u_{th}\right)+\gamma_{i}^{t+1,n}\tau \end{array}$$

where the first component on the right side of the equations is contributed by the errors propagated spatially from the upper layer, and the second component is due to the temporal error backpropagation. Clearly, the potential error needs to be propagated backward in space and time. Different surrogate gradient functions were proposed to solve the discontinuity issue with the firing function in Equation (2) (Wu et al., 2018). A rectangular function is proven to be effective and simple, and thus the gradient function can be approximated by


(6) $$\begin{array}{l} {\text{Θ}}^{{}^{\prime }}\left(u\right)\approx \frac{1}{a}sign\left(|u-u_{th}|<\frac{a}{2}\right) \end{array}$$

where *sign* (·) is the sign function, and *a* is the width of the non-zero region. In TBPTT, weight gradients are accumulated over the truncation interval by summing up all the gradients computed at each time step, expressed as


(7) $$\begin{array}{l} \begin{array}{c} \frac{\partial L}{\partial w_{ij}^{n}}=\underset{t}{\sum }\gamma_{i}^{t,n}s_{j}^{t,n-1} \end{array} \end{array}$$

where the summation goes over the truncation interval. With the computed gradients, weight parameters can be updated by an optimization method, such as stochastic gradient descent (SGD) and Adam (Sutskever et al., 2013; Kingma and Ba, 2015). The implementation details of the training algorithm are explained in Algorithm 1.

**Algorithm 1:** Training algorithm for one batch iteration.

**Inputs:** Network inputs ${\left\{X^{t}\right\}}_{t=1}^{T}$, class labels Y, parameters and initial neural states of the main network ${\left\{W^{l},U^{t_{0},l},\;S^{t_{0},l}\right\}}_{l=1}^{N_{L}}$, parameters and initial neural states of local classifiers ${\left\{W_{c}^{l},U_{c}^{t_{0},l},\;S_{c}^{t_{0},l}\right\}}_{l=1}^{N_{c}}$, training window *T*, training variables (*k, n*), other hyper-parameters.
Initialize all the parameters and neural states.
for interval *i* = 1 *to T*/*k* do
**Forward pass:**
for time τ = 1 *to k* do
*t* = τ+(*i*− 1)^*^k
for layer *l* = 1 *to N*_*L*_ do
Network state update: {*U*^*t,l*^, *S*^*t,l*^}← Update{*W*^*l*^, *U*^*t*−1, *l*^, *S*^*t,l*−1^, *S*^*t*−1, *l*^}, **Eq**. (1), (2).
if *l % n* = = 0,
Classifier state update: $\left\{U_{c}^{t,l},\;S_{c}^{t,l}\right\}\leftarrow$ Update$\left\{W_{c}^{l},U_{c}^{t-1,l},\;S_{c}^{t,l-1},S_{c}^{t-1,l}\right\}$, **Eq**. (1), (2).
end if
end for
end for
for layer *l* = 1 *to N*_*L*_ do
if *l % n* = = 0,
Compute loss: *L*← Loss function$\left\{Y,\;\underset{t}{\sum }S_{c}^{t,l}\right\}$, **Eq**. (3)
end if
end for
**Backward pass:**
for time τ = *k to* 1 do
*t* = τ+(*i*− 1)^*^k
for layer *l* = *N*_*L*_ *to* 1 do
if *l % n* = = 0,
Compute errors and gradients at classifiers.
Accumulate gradients.
end if
Backpropagate errors and compute gradients: **Eq**. (4), (5), (7)
Accumulate gradients.
end for
end for
Update weights.
end for

## 3. Experiments

### 3.1. Experiment setup

We evaluated the proposed training algorithm on four different spiking convolutional neural networks (SCNNs) adapted from LeNet, AlexNet, VGG11, and ResNet18 architectures (Lecun et al., 1998; Karen Simonyan, 2014; Krizhevsky et al., 2017; Amir et al., 2021). The networks were tested on two different types of datasets: static frame-based datasets and dynamic event-based datasets. LeNet was used to classify extended MNIST (EMNIST) dataset (Cohen et al., 2017) and DvsGesture dataset (Amir et al., 2017), while AlexNet was used to classify CIFAR10 dataset (Krizhevsky, 2009) and CIFAR10-DVS dataset (Li et al., 2017). In particular, to verify the scalability of the proposed method, deep networks such as VGG11 and ResNet18 were used to perform more complex tasks, classifying N-Caltech101 (Orchard et al., 2015) and a tiny version of the Es-ImageNet (Lin et al., 2021), respectively. The Tiny-Es-ImageNet contains 100 K samples of a resolution of 64 × 64 with 200 classes. Simulations were performed using PyTorch framework (Paszke, 2019). The mean squared error (MSE) loss function and SGD optimization method with momentum were used for training LeNet and AlexNet (Sutskever et al., 2013). The CrossEntropy loss function and Adam optimizer were applied to train VGG11 and ResNet18 because of the task complexity. As for regularization, a dropout layer was added after each convolutional or fully-connected layer (Srivastava et al., 2014). Accuracy results were recorded after 100 training epochs for all the simulations. A step-decay scheduling method was used to reduce learning rate by a factor of 2 every 20 epochs. The other hyper-parameters used in all the experiments are listed in Table 1. We varied the values of (*k, n*) and obtained corresponding classification accuracy on each dataset.

**Table 1:** Hyperparameter setting.

**Hyperparameter**	**EMNIST**	**DvsGesture**	**CIFAR10**	**CIFAR10-DVS**	**N-Caltech101**	**Es-ImageNet**
Batch size	1,024	32	128	128	128	512
Momentum	0.9	0.9	0.9	0.9	-	-
Time steps, *T*	20	60	10	100	60	8
Gradient width, *a*	0.5	0.5	0.5	0.5	0.5	0.5
Dropout rate	0	0–0.2	0–0.15	0–0.2	0–0.3	0
Learning rate	0.2–0.5	0.005–0.2	0.1–0.2	0.1–0.5	0.0001–0.0005	0.0001–0.001
Leaky constant, **τ**	0.9	0.3	0.8	0.8	0.5	0.5
Threshold potential, **u**_**th**_	0.4–0.6	0.2–0.5	0.3–0.5	0.3–0.6	0.5	0.5–0.8

### 3.2. Spiking convolutional neural networks

The network structures used in the experiments are listed in Table 2. The spiking LeNet is a five-layer spiking CNN adapted from the original LeNet, consisting of three convolutional layers, two average-pooling layers, and two fully-connected layer. A LIF neuron layer is placed after each of these layers, so that each layer outputs spikes. We used two network scales. The smaller network, LeNet-1, with a smaller number of channels and neurons, was tested on EMNIST, while the larger network, LeNet-2, was for DvsGesture because of pattern complexity. We also constructed a nine-layer spiking AlexNet with similar network settings to the original AlexNet. It consists of six convolutional layers followed by two average-pooling layers, and three fully-connected layers. The spiking VGG11 is adapted from the original VGG11, consisting of eight convolutional layers and three fully-connected layers. The spiking ResNet18 adopts the spike-element-wise ResNet block structure proposed in Fang et al. (2021), which guarantees the identity mapping property. For both VGG11 and ResNet18, A batch normalization layer is inserted after each trainable layer.

**Table 2:** Network architectures.

**Network**	**Architecture**
LeNet-1	6C5-6AP2-16C5-16AP2-120C5-128FC-47FC
LeNet-2	64C5-64AP2-128C5-128AP2-128C5-256FC-11FC
AlexNet	96C3-256C3-256AP2-384C3-384AP2-512C3-384C3-256C3-4096FC-1024FC-10FC
VGG11	32C3-32AP2-64C3-64AP2-128C3 × 2-128AP2-256C3 × 2-256AP2-512C3 × 2-512AP2-1024FC-1024FC-101FC
ResNet18	32C3-32RB × 2-64RB × 2-128RB × 2-256RB × 2-Adaptive AP-200FC, RB: C3 × 2

C, AP, and FC represent a convolutional layer, an average pooling layer, and a fully-connected layer, respectively. RB is a ResNet block. For VGG11 and ResNet18, batch normalization is applied after each convolutional layer and FC layer.

### 3.3. Encoding methods

#### 3.3.1. Frame-based datasets

Since the images from both EMNIST and CIFAR10 datasets are comprised of integer-valued pixels, they are incompatible with SNNs. The widely used conversion method is rate encoding, which converts each pixel into a spike train with a frequency proportional to the pixel intensity. This method suffers from high training latency and precision loss. Many works proposed a direct encoding method that uses the first layer as an encoding layer, directly receiving intensity values and outputting spikes as inputs to the next layer (Esser et al., 2016; Wu et al., 2019; Deng et al., 2020). This method largely reduces training latency and retains good performance. Although the first layer computes as an ANN layer, under the fact that networks generally consist of tens or hundreds of layers, this has little impact on the computational efficiency of SNNs. Thus, we adopted the direct encoding method in our experiments.

#### 3.3.2. Event-based datasets

DVS cameras produce event streams encoded in timestamps, xy coordinates, and polarity. SNNs cannot directly process the raw data. We converted each encoded event streams into a time series of event images with two channels and binary pixel intensity. The two channels correspond to the polarity of events, and the binary intensity indicates the occurrence of an event at the pixel location. Due to long recording time and high resolution, we accumulated all the event images in a defined time window Δ*t* into one new event image and took the first *T* new images as inputs to SNNs. The values of (Δ*t,T*) are (20 *ms*, 60) for DvsGesture dataset, (5 *ms*, 100) for CIFAR10-DVS dataset, and (5 *ms*, 60) for N-Caltech101 dataset, respectively. Different from DVS-recorded datasets, ES-ImageNet was converted from the whole ILSVRC2012 ImageNet dataset by using the Omnidirectional Discrete Gradient (ODG) algorithm similar to the DVS recording mechanism (Lin et al., 2021). Since each event sample has only 8 time steps, no conversion was needed.

### 3.4. Classification accuracy results

Temporal truncation ignores the temporal dependency spanning across truncated intervals in the backward pass, which introduces bias on short-term dependency. The local training method utilizes local errors to learn features that only benefit advantages, these two methods could have a negative impact on classification performance. Thus, it is necessary to investigate and analyze their roles. In Section 2.2, we defined a variable pair (*k, n*), as the length of the truncated temporal interval and the number of layers in a local block, respectively. The case where *k* equals the total time step *T* refers to the standard BPTT, whereas *k* = 1 suggests no temporal dependency in the backward pass. In this section, we experiment with different sets of (*k, n*) in each classification task and analyze the change of classification accuracy.

**Figure 3:**
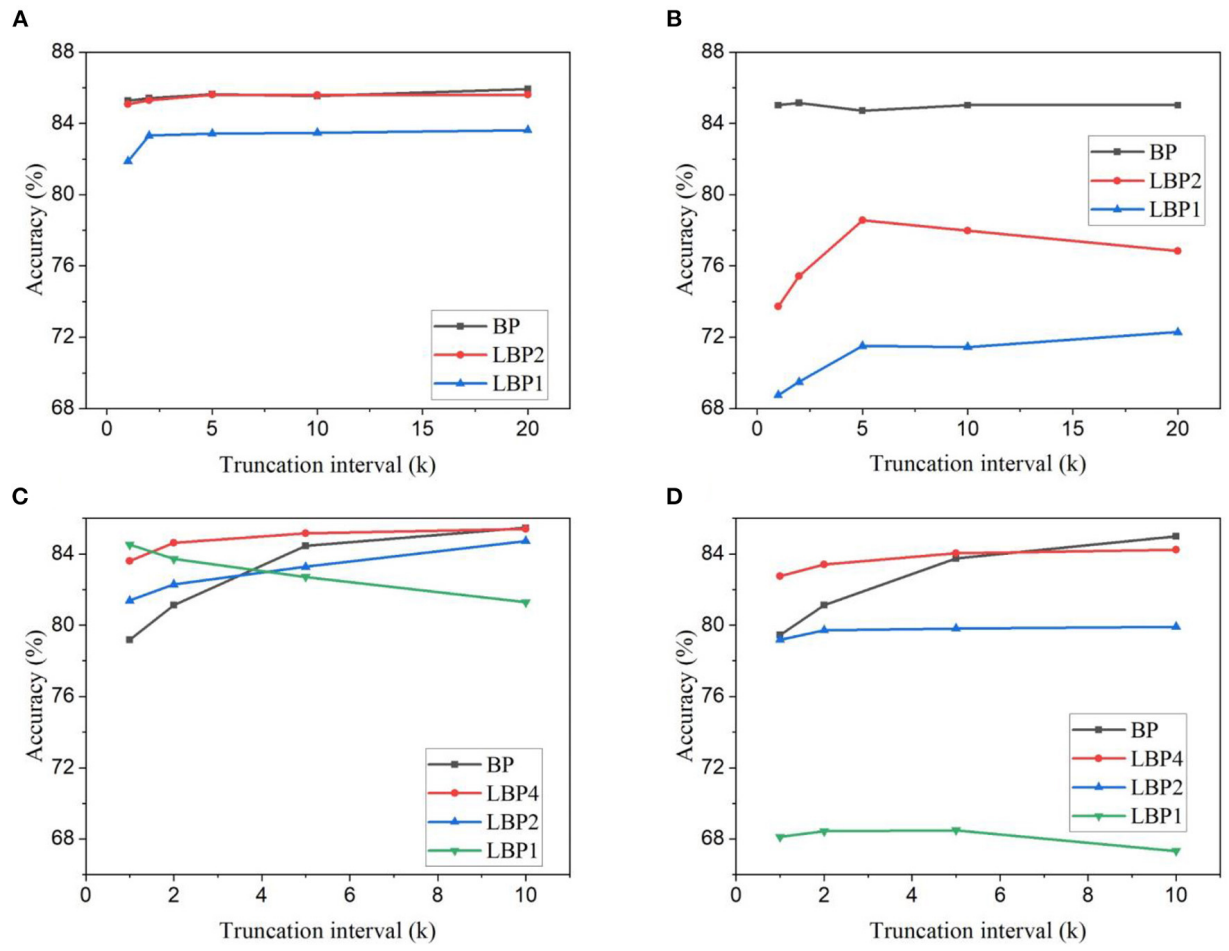
Classification accuracy for different truncation intervals and local block size. Accuracy results are obtained for LeNet-1 on EMNIST dataset with **(A)** trainable classifiers and **(B)** random classifiers. And accuracy results are obtained for AlexNet on CIFAR10 dataset with **(C)** trainable classifiers and **(D)** random classifiers. In LBPn, n indicates the number of layers in a local block.

For classification on EMNIST dataset, *k* was chosen from the set {20, 10, 5, 2, 1}, and *n* from the set, {4, 2, 1}. For classification on CIFAR10 dataset, *k* was chosen from the set {10, 5, 2, 1}, and *n* from the set, {8, 4, 2, 1}. The accuracy results on the two datasets are shown in Figure 3, respectively. Since the spiking LeNet is of four layers, *n* = 4 corresponds to the standard BP. And *n* = 8 in AlexNet also corresponds to the standard BP. We use *LBPn* to indicate the local BP with *n* layers in each local block. From the results, for both LeNet and AlexNet, accuracy tends to decrease with the truncation interval in most cases, especially when the interval is small. The same behavior is also observed in the case of AlexNet classifying EMNIST. This reveals that temporal truncation has a negative impact on the accuracy regardless of the network size. In the classification experiment on EMNIST, local learning methods are affected by temporal truncation more significantly than standard BP. BP shows the best accuracy compared against LBP2 and LBP1, which suggests that LBP causes the loss of useful information. However, in the classification experiment on CIFAR10, BP is more severely affected by temporal truncation with a 6.30% accuracy drop compared with the maximum drop of 3.35% for LBPs, as indicated in Figure 3C. LBP4 shows the best results in most cases. This can be due to that LBP alleviates the overfitting effect (Belilovsky et al., 2020). LBP divides the network into smaller blocks and trains each block separately for the same task. In some way, the actual number of parameters required to learn features for the task is reduced, which leads to less severe overfitting. The combined effect of temporal truncation and local training can be observed in Figures 3C, D, where the accuracy of BP is affected by temporal truncation more severely than LBPs. LBP2 has lower accuracy at large intervals, but gets closer to and even surpasses BP when the interval shrinks. In the case of LBP1, the accuracy is improved all the way. Comparing the results obtained from trainable classifiers and random classifiers, we found that LBP with random classifiers results in a more substantial accuracy drop, as shown in Figures 3B, D. For example, in Figure 3B, at *k* = 20, applying random classifiers in LBP1 causes a 14.91% accuracy drop, whereas only 0.91% is incurred for BP.

**Figure 4:**
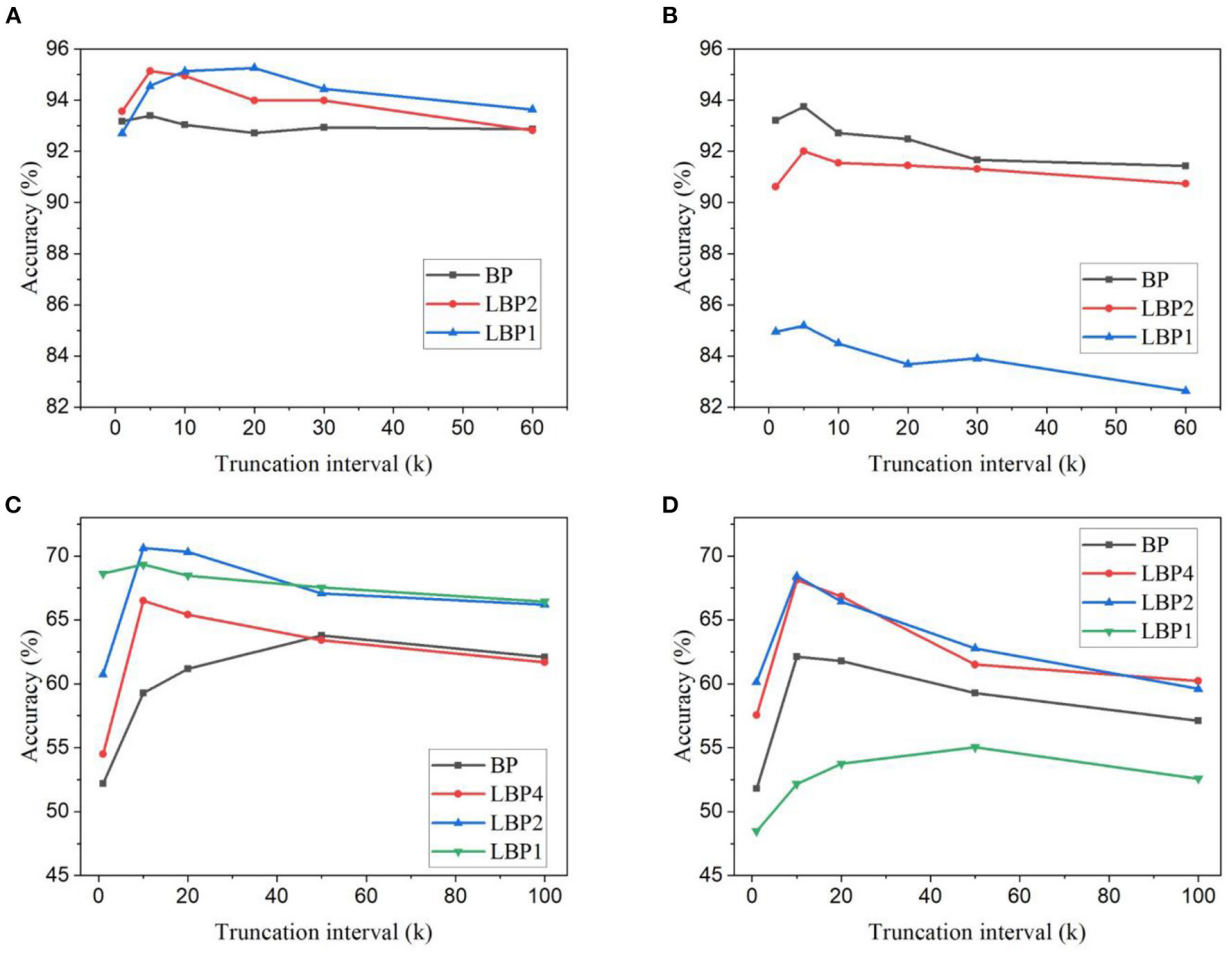
Classification accuracy for different truncation intervals and local block sizes. Accuracy results are obtained for LeNet-2 on DvsGesture dataset with **(A)** trainable classifiers and **(B)** random classifiers. And accuracy results are obtained for AlexNet on CIFAR10-DVS dataset with **(C)** trainable classifier and **(D)** random classifier.

In the simulations on DvsGesture dataset, *k* was chosen from the set, {60, 30, 20, 10, 5, 1}, and *n* from the set, {4, 2, 1}. In the simulations on CIFAR10-DVS dataset, *k* was chosen from the set, {100, 50, 20, 10, 1}, and *n* from the set, {8, 4, 2, 1}. The accuracy results of the two datasets are shown in Figure 4. Different from the frame-based datasets, the DVS-recorded datasets show different changes of accuracy with temporal truncation. The accuracy tends to increase while the truncation interval decreases and then decreases when the interval becomes small. This phenomenon could be because temporal truncation with a large *k* generally gives better convergence at the cost of a long training time, while a small *k* can cause the network not to converge, thus resulting in bad performance (Aicher et al., 2019). Due to a long temporal update window, the vanishing gradient problem could appear in the BPTT algorithm. Based on the backward update formulas in Equations (4) and (5), in the temporal dimension, the gradients decay by a factor of (τ−θΘ′) for each time step. For example, if τ = 0.8 and θ = 0.3, after 10 steps, the rate lies in the range [6*e*^−6^, 0.1], depending on the binary spike gradients Θ′. This means that after only 10 steps, the temporal gradients would become very small and gradually disappear with more steps. A long update window cannot make effective use of the temporal gradients. Truncating the long window into small update intervals avoids the vanishing of temporal gradients and makes better use of temporal connections, hence improving network performance. However, a very small truncation window cannot capture the temporal dependency that spans a larger range in the input. Thus, there exists an optimal truncation length that can lead to the best accuracy. This suggests that trained on the datasets containing temporal information, SNNs can benefit from temporal truncation in improving classification performance. But the optimal truncation interval varies dependent on datasets. Moreover, as shown in Figure 4A, on DvsGesture dataset, LBP leads to better accuracy than BP in the case of trainable classifiers. The same comparison can be observed in the results of CIFAR10-DVS dataset, as shown in Figures 4C, D. This further confirms that LBP could reduce overfitting effect because both DVS-recorded datasets contain a small number of training samples. The combining effect can be seen in Figures 4A, C, D, where the improvement in LBPs is more significant than in BP. Additionally, as shown in Figures 4B, D, applying random classifiers in LBP1 incurs significant accuracy loss, namely, 10.99% on DvsGesture and 13.86% on CIFAR10-DVS, when *k* is the total time step.

**Figure 5:**
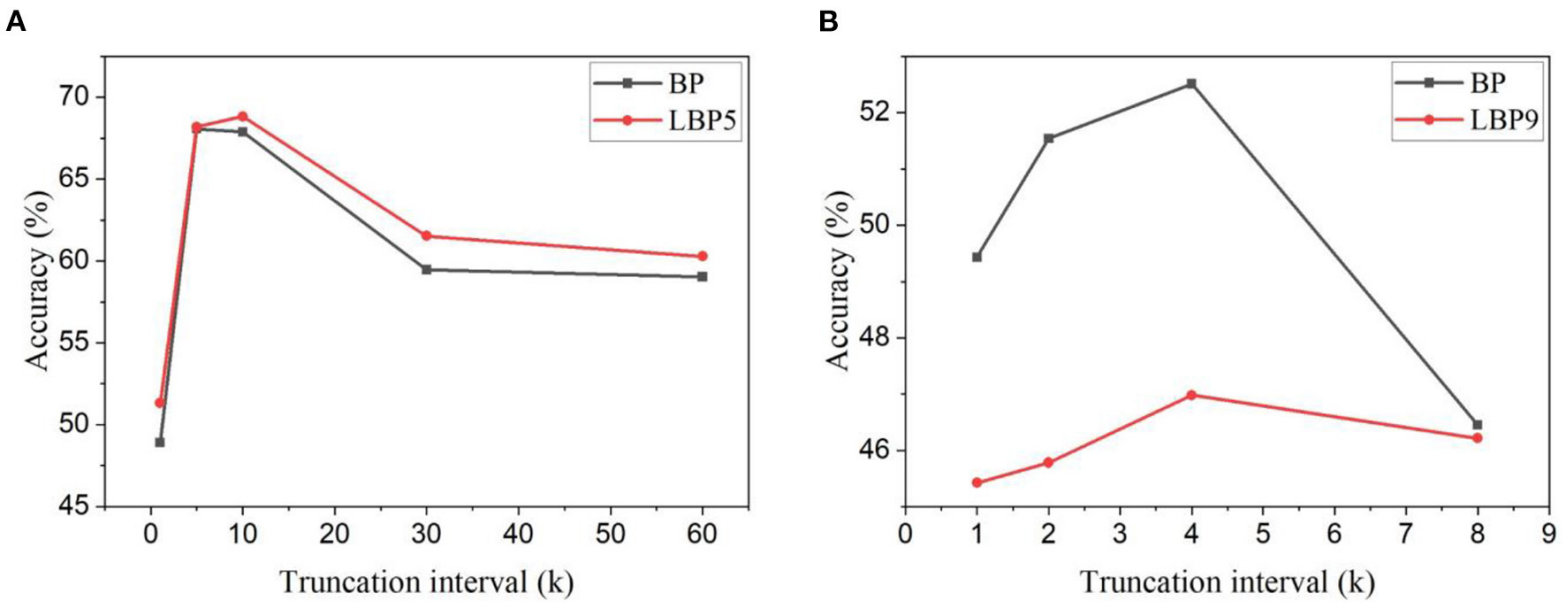
Classification accuracy for different truncation intervals and local block size. **(A)** Accuracy results were obtained for VGG11 on the N-Caltech101 dataset. **(B)** Accuracy results were obtained for ResNet18 on the Tiny-Es-ImageNet dataset.

To further verify the scalability, the proposed algorithm was also investigated in deeper networks for more complex tasks, namely, N-Caltech101 and Es-ImageNet. The accuracy results are shown in Figure 5. The same phenomenon on the change of accuracy with the truncation interval can be observed for both BP and LBP. These results further confirm that temporal truncation exhibits the same effect on classification accuracy on event-based datasets. A very small or long interval leads to lower accuracy. For VGG11, an optimal interval can be seen at 5 and 10 for BP and LBP5, respectively, as shown in Figure 5A. LBP is able to improve the accuracy. For ResNet18, the optimal interval can be seen as 4 for both BP and LBP, as shown in Figure 5B. However, in this case, LBP cannot improve the accuracy, which can be attributed to that in the deep network architecture, due to high dataset complexity, the information loss caused by local training exceeds the benefits of overfitting reduction.

In summary, temporal truncation affects the classification performance of SNNs differently on different types of datasets. It shows an overall negative impact on classifying frame-based datasets, whereas it brings on benefits in improving classification performance on DVS-recorded datasets with optimally chosen intervals. From all the experiments, we can see that a good choice of the truncation interval appears in the range from 2 to 10. Regarding local training, in the scenario where overfitting is not severe, such as in LeNet-1 trained on EMNIST, LBP causes inferior performance. On the other hand, LBP can alleviate overfitting effect to some extent and lead to better accuracy. The roles of temporal truncation and local training are not orthogonal. Instead, in most cases, they tend to function synergistically. In addition, the use of random classifiers results in accuracy loss, especially in LBP1.

## 4. Computational cost

DNNs are typical of tens of or hundreds of layers with millions of or even billions of parameters. They are both computationally and memory intensive, making them difficult to be implemented in hardware. Training DNNs poses a great challenge in hardware design. Thus, it is essential to assess the computational cost of a training algorithm. In this section, we will analyze the computational cost of the proposed training method on different hardware platforms in terms of required GPU memory, external memory access, and number of arithmetic operations.

### 4.1. GPU memory cost

Temporal truncation reduces the length of backward pass and eliminates the requirement to store the history of neural states before the current interval, which leads to memory saving. Local training avoids the necessity to build a whole backward computational graph by training SNNs block by block, which also leads to memory saving. In this section, we measure and compare the maximum GPU memory used to perform each classification task under different settings of (*k, n*) in Pytorch. The measurement was done with the commonly-used command *max_memory_allocated* in Pytorch (Li G. et al., 2021; Wang et al., 2021).

**Figure 6:**
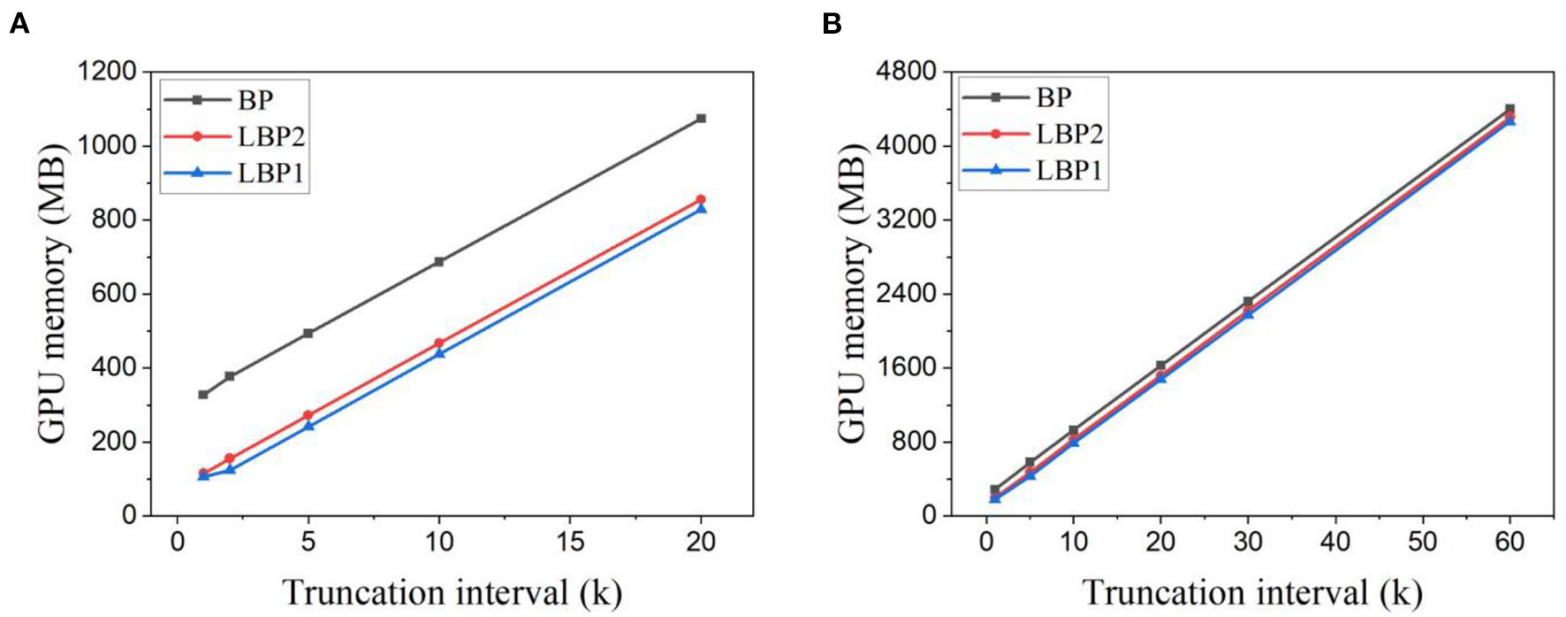
GPU memory cost measured in Pytorch for training **(A)** LeNet-1 on EMNIST dataset and **(B)** LeNet-2 on DvsGesture dataset. Both SNNs were trained with trainable classifiers. The batch size is 1,024 and 32 for EMNIST dataset and DvsGesture dataset, respectively.

Figure 6 shows the measured GPU memory cost for training LeNet with trainable classifiers on different datasets. Due to the larger network scale and longer training window, the memory cost of LeNet-2 is much higher than that of LeNet-1. Clearly, memory cost decreases linearly with decreasing truncation interval. Compared to BP, LBP2 can reduce memory cost. As shown in Figures 6A, B, the reduction percentage increases with decreasing interval from 20.14 to 64.73% for classifying EMNIST and from 2.06 to 32.61% for classifying DvsGesture. Further decreasing *n* in LBP contributes to minor change. This is mainly because the uneven distribution of layer neurons and parameters in networks causes particular layers to dominate in memory occupation. Replacing trainable classifiers with random classifiers has a negligible effect on reducing memory cost.

**Figure 7:**
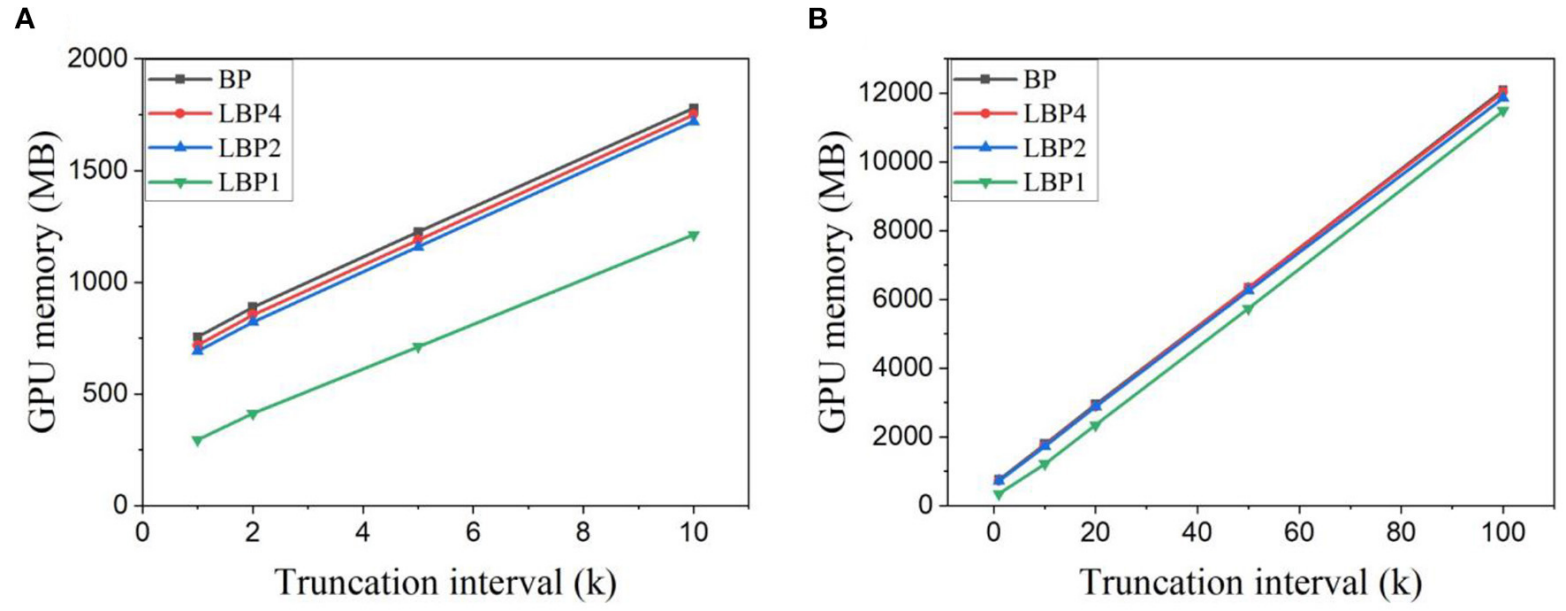
GPU memory cost measured in Pytorch for training AlexNet on **(A)** CIFAR10 dataset and **(B)** CIFAR10-DVS dataset. Trainable classifiers are used. The batch size is 128 for both cases.

The same observations can be seen in the memory cost for training AlexNet on CIFAR10 and CIFAR10-DVS datasets, as shown in Figures 7A, B, respectively. LBP1 helps reduce the memory cost from 31.86 to 60.91% for classifying CIFAR10, and from 3.44 to 54.42% for classifying CIFAR10-DVS with the decreasing truncation interval. Using random classifiers leads to less than a 2% reduction.

In the simulation framework, the memory cost of neural networks are caused by the storage of parameters, network states, and computational graphs (CGs) (Gao et al., 2020). The memory cost (MC) of an SNN can be determined by


(8) $$\begin{array}{l} MC=N\left(w\right)+MC\left(CG\right)+C=N\left(w\right)+N\left(states\right)+N\left(inter.\right)\;\\ \;+N\left(grads\right)+C \end{array}$$

where *N*(*w*), *N*(*states*), *N*(*inter*.), and *N*(*grads*) are the number of trainable parameters, neural states, intermediate tensors allocated in computational graphs, and gradients, respectively, *C* is a constant representing the memory consumed by CUDA workspace. In TBPTT, networks are unfolded in time, and the history of the states, intermediate tensors, and gradients are saved. Once the backward update is finished, all the history is discarded. Thus the memory cost of TBPTT with a truncation interval k is


(9) $$\begin{array}{l} MC=k\left(MC\left(CG\right)\right)+N\left(w\right)+C \end{array}$$

The benefit of local training methods is only possible at the last time step of the truncation interval at which a partial graph of the block length is saved. When local training method is applied, the memory cost becomes


(10) $$\begin{array}{l} MC=\left(k-1\right)\left(MC\left(CG\right)\right)+MC_{Local}\left(CG\right)+N\left(w\right)+C \end{array}$$

where *MC*_*Local*_(*CG*) is the maximum memory cost of local training methods determined by layer distribution in the network. *MC*(*CG*) depends on network architectures and remains constant in time, which explains the linearity in the memory change. The memory gap between BP and LBP is determined by the difference between *MC*(*CG*) and *MC*_*Local*_(*CG*), i.e., the difference between a whole graph and a partial graph. The difference is the function of network architectures and proportional to the block length, remaining unchanged with *k*.

### 4.2. Memory access and arithmetic operations analysis

The BP algorithm requires hardware to store all the neural states at each layer before performing backward updates from the top layer, as those states are needed to compute errors and gradients along the backward pass. More costly, the BPTT algorithm introduces an extra time dimension and requires the storage of the whole history of all the states at each layer. General hardware, such as CPU, GPU, and field-programmable gate array (FPGA), has limited on-chip memory capacity, which is not enough to accommodate the states and parameters of state-of-the-art networks. The intermediate states and network parameters have to be saved in external memories such as DRAM. Thus, the BPTT algorithm adds a significant memory overhead and a huge data communication burden on hardware. Frequent communication also brings on high energy consumption since memory access consumes much more energy than arithmetic operations. For example, for the 45 nm CMOS process, memory access consumes 3 orders of magnitude more energy (Han et al., 2015). Reducing memory access frequency can lead to significant energy and time saving. In this section, we analyze and model the memory traffic pattern and the number of arithmetic operations in training SNNs with the proposed training algorithm.

**Figure 8:**
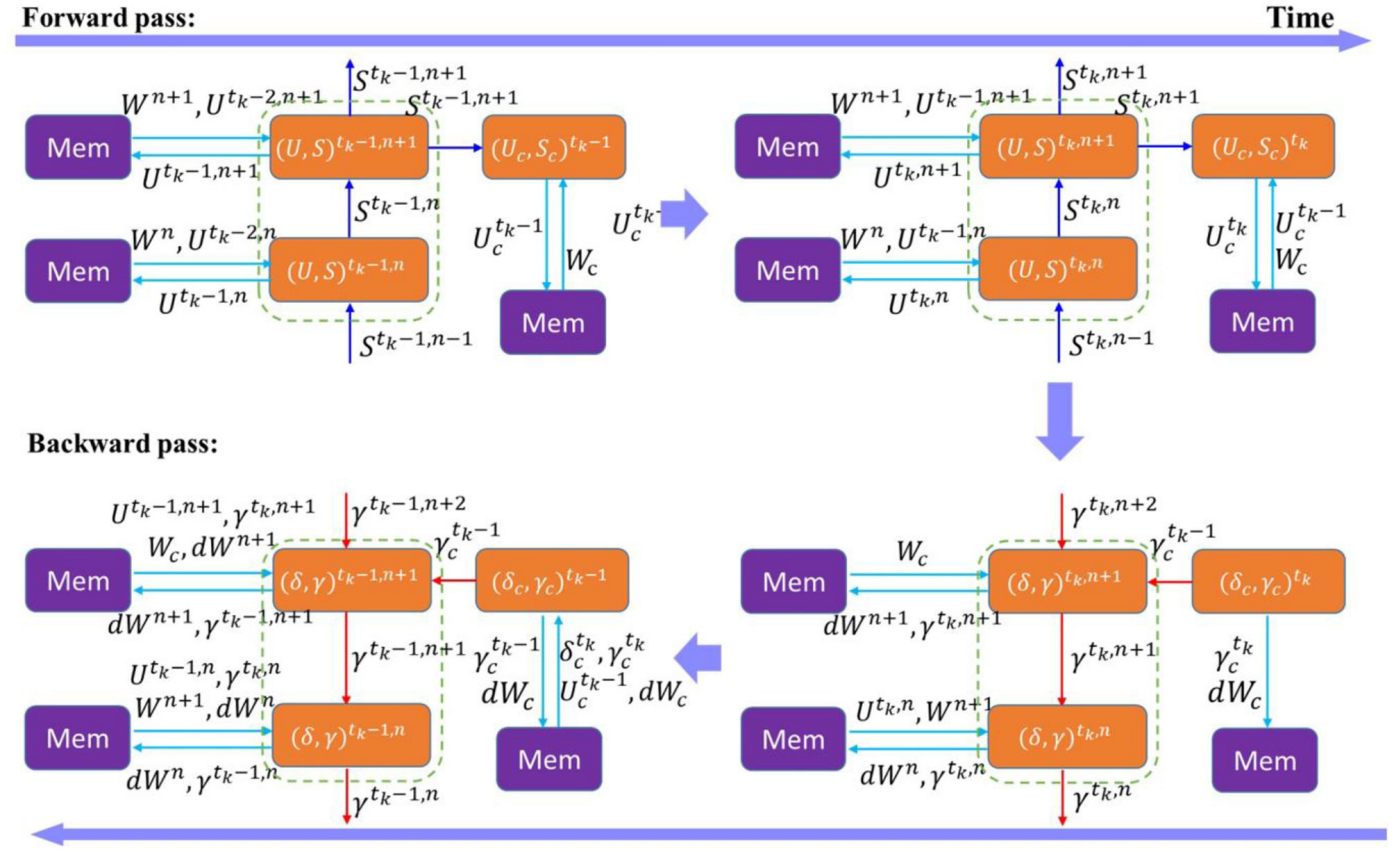
Memory traffic of forward pass and backward pass in a local learning block during a truncation interval. (*U, S*)^*t,n*^ represents the neural states in the layer n of the main network at time t. *W*^*n*^ and *dW*^*n*^ are the weights and gradients in the layer n. (δ, γ)^*t, n*^ represents the spike error and potential error in the layer n at time t. The parameters and states of the local classifier are indicated by the subscript c. *t*_*k*_ is the end of the truncation interval.

Figure 8 illustrates the data transfer between external memories and processing cores in both forward pass and backward pass of a local block during a truncation interval. Assume that on-chip memory has the capacity to store the parameters and batch neural states of a layer. During forward update, at time *t*, each layer has to read its weights *W*^*n*^ and previous neural states *U*^*t*−1, *n*^ from an external memory, and write the updated states *U*^*t,n*^ back to the external memory in separate locations. We omit the transfer of spikes since they are one-bit data. The number of read and write operations in forward pass is expressed by


(11) $$\begin{array}{l} \begin{array}{c} N_{r}^{f}=\overset{N_{l}}{\underset{n=1}{\sum }}\left(\left|W^{n}|+|U^{n}\right|\right)+\left(\left|W_{c}|+|U_{c}\right|\right),\;\\ N_{w}^{f}=\overset{N_{l}}{\underset{n=1}{\sum }}|U^{n}|+|U_{c}|\; \end{array} \end{array}$$

where *N*_*l*_ is the number of layers in a local block, |*W*^*n*^| and |*W*_*c*_| are the total number of weights in the layer *n* of the main network and weights in the classifier layer, respectively, |*U*^*n*^| and |*U*_*c*_| are the total number of batch neural states in the layer *n* and the classifier layer, respectively. During backward update, the network needs to compute the errors (δ, γ)^*t,n*^, in each layer at each time step, and propagate the potential error backward through layers and time. At any time step in the middle of truncation interval, for example, at *t*_*k*_−1 in Figure 8, to compute the errors at the layer *n*, the network has to read the current neural states $U^{t_{k}-1,\;n}$, weights from the upper layer *W*^*n*+1^, and potential errors from the next time step $\gamma^{t_{k},n}$. Gradients *dW*^*n*^ also need to be read for accumulation. The updated potential errors $\gamma^{t_{k}-1,n}$ and gradients *dW*^*n*^ are written back to memory. The number of read and write operations in the middle of the backward pass is expressed by


(12) $$\begin{array}{l} N_{r}^{b}=\sum_{n=1}^{N_{l}}\left(\left|W^{n}\right|+2\left|U^{n}\right|\right)+\sum_{n=1}^{N_{l}-1}\left(\left|W^{n+1}\right|\right)+2\left|W_{c}\right|\;\\ \;+3\left|U_{c}\right|\; \end{array}$$

(13) $$\begin{array}{l} N_{w}^{b}=\sum_{n=1}^{N_{l}}\left(\left|W^{n}\right|+\left|U^{n}\right|\right)+\left|W_{c}\right|\;\\ \;+\left|U_{c}\right|\;\; \end{array}$$

We simplify the formulation using the fact that |*dW*^*n*^| = |*W*^*n*^| and |γ^*n*^| = |*U*^*n*^|. Clearly, in the middle of the backward pass, the local training method does not help reduce the number of memory access because the consecutive execution between the forward update and backward update is prohibited at the time step. At the end of the interval where *t* = *t*_*k*_, there is no temporal component needed to compute the errors. Since the backward pass of the block can immediately start after the forward pass finishes, the current neural states of the top layer and classifier can be buffered on the chip for the backward update, eliminating the need to read them from external memory. There is also no need to read the gradients. Thus, the number of reads can be reduced to


(14) $$\begin{array}{l} N_{r}^{b}=\overset{N_{l}-1}{\underset{n=1}{\sum }}\left(\left|W^{n+1}|+|U^{n}\right|\right)+|W_{c}| \end{array}$$

Using random weights in the classifier can further reduce memory access volume by removing all the operations on classifier weights. As argued in Mostafa et al. (2018), random weights can be generated on the fly by random number generators (RNGs), avoiding the storage in external memories. It could cause either resource overhead in the case of multiple RNGs for parallel processing, or increased latency as computation needs to wait for the generation of random weights. Also, on-chip memory has to be allocated to hold them before computation finishes.

SNNs replace MAC operations with multiplex-accumulation operations through event communication in the forward update. However, in the backward update, full-precision errors are the information carriers, so MAC operations are inevitable, as indicated in Equation (4). The addition operations in the forward update are dominated by the computation of synaptic input, which is proportional to the size of weight matrices and input spike sparsity, expressed by


(15) $$\begin{array}{l} N_{add}^{f}=\overset{N_{l}}{\underset{n=1}{\sum }}\alpha^{n-1}M^{n}+\frac{\alpha_{c}|W_{c}||U_{c}|}{N_{c}} \end{array}$$

where α^*n*−1^ is the input sparsity to the layer *n*, *N*_*n*_ and *N*_*c*_ are the number of neurons in the layer *n* and a classifier, respectively, and $C_{o}^{n}$ is the number of output channels in a convolutional layer *n*. *M*^*n*^ is the total number of additions without considering sparsity, computed by $\left(\left|W^{n}||U^{n}\right|\right)/C_{o}^{n}$ for a convolutional layer and $\left(\left|W^{n}||U^{n}\right|\right)/N_{n}$ for a fully-connected layer. In the backward update, according to Equations (4), (5), and (7), the number of additions is estimated as


(16) $$\begin{array}{l} N_{add}^{b}=\overset{N_{l}}{\underset{n=1}{\sum }}\left(2|U^{n}|+N_{b}\alpha^{n-1}|W^{n}|\right)\;+2|U_{c}|+N_{b}\alpha_{c}|W_{c}| \end{array}$$

It is worth to note that the batch size *N*_*b*_ is multiplied with the weight matrix size representing a computation of a batch of gradients. MAC operations only appear in Equation (4), which are used to propagate errors backward from upper layers. The number of MACs can be expressed as


(17) $$\begin{array}{l} N_{mac}^{b}=\overset{N_{l}-1}{\underset{n=1}{\sum }}M^{n+1}+\frac{\left|W_{c}||U_{c}\right|}{N_{c}} \end{array}$$

### 4.3. Memory access

**Figure 9:**
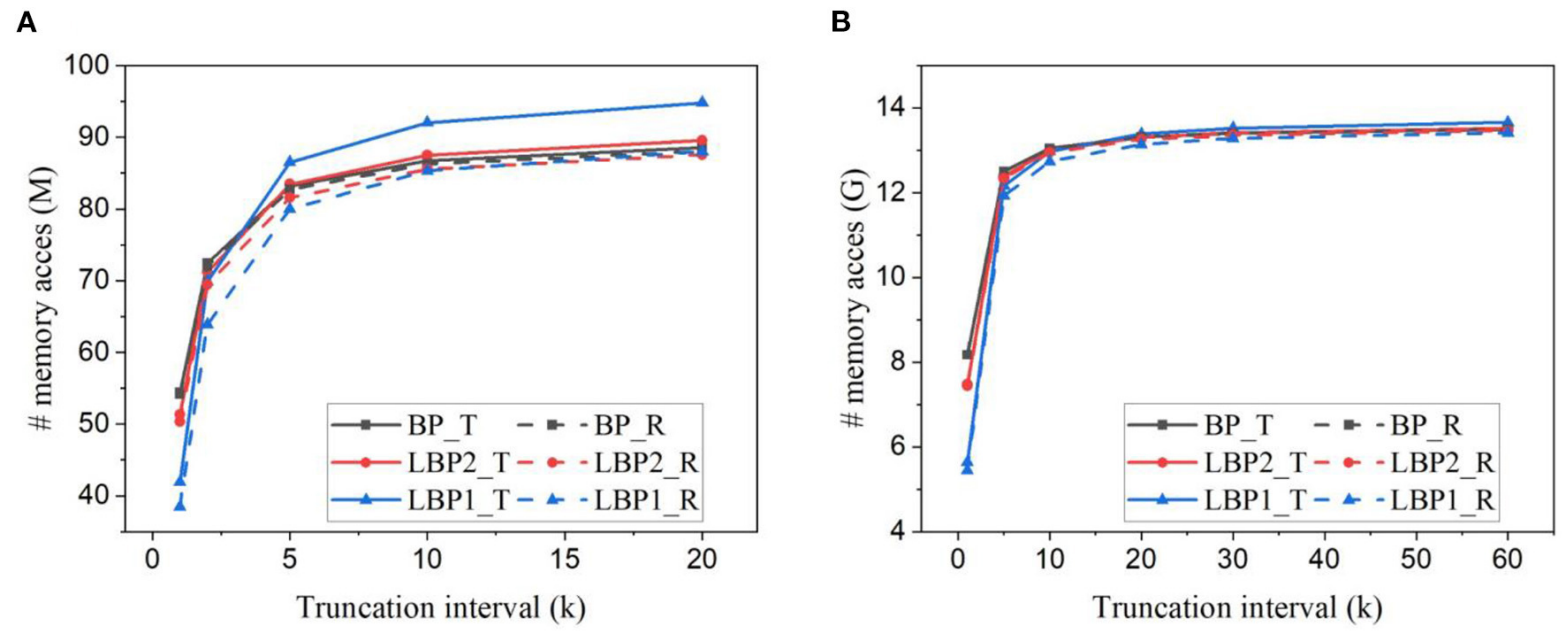
Number of memory access estimated for training **(A)** LeNet-1 on EMNIST dataset and **(B)** LeNet-2 on DvsGesture dataset for one batch iteration. Solid lines and dashed lines represent the results obtained with trainable (T) classifiers and random (R) classifiers, respectively. The batch size is 128.

We estimated the number of memory access, including reads and writes required in one training iteration. The batch size is kept as 128 for all the cases. Figures 9A, B show the estimation for training LeNet with both types of classifiers on EMNIST dataset and DvsGesture dataset, respectively. The number of memory access decreases with the truncation interval, rapidly when the interval becomes small. With trainable classifiers, LBPs lead to more memory access when the interval is large, because of the overhead of classifier weights. When the interval is small, the advantage of LBPs becomes more significant, thus overcoming the overhead. On the contrary, the use of random classifiers avoids the overhead, making LBPs better than BP at all intervals. Specifically, on EMNIST dataset, temporal truncation can contribute to around a 55% reduction for LBP1 with either type of classifiers. LBP1 can lead to around 23% reduction with trainable classifiers and 29% reduction with random classifiers at *k* = 1 against BP, respectively.

**Figure 10:**
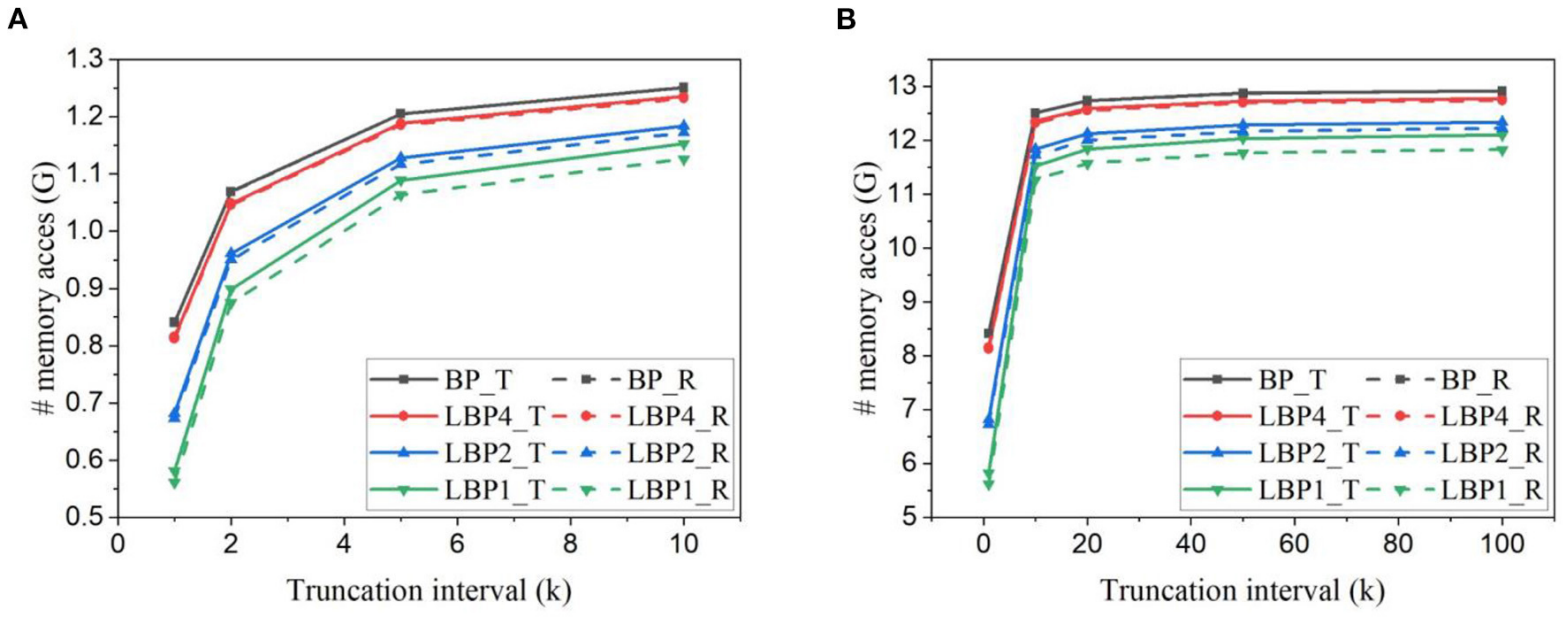
Number of memory access estimated for training AlexNet **(A)** on CIFAR10 dataset and **(B)** on CIFAR10-DVS dataset.

The estimated number of memory access for training AlexNet on CIFAR10 dataset and CIFAR10-DVS dataset is shown in Figures 10A, B. In AlexNet, the size of a classifier layer is much smaller compared to the network layers. The small overhead of trainable classifiers is overcome by the benefit. So LBPs lead to a reduction in memory access at all intervals. On CIFAR10, temporal truncation can lead up to around 50% reduction in LBP1. Compared against BP, LBP1 can lead up to 31% with trainable classifiers and 33% with random classifiers, respectively.

### 4.4. Arithmetic operations

**Figure 11:**
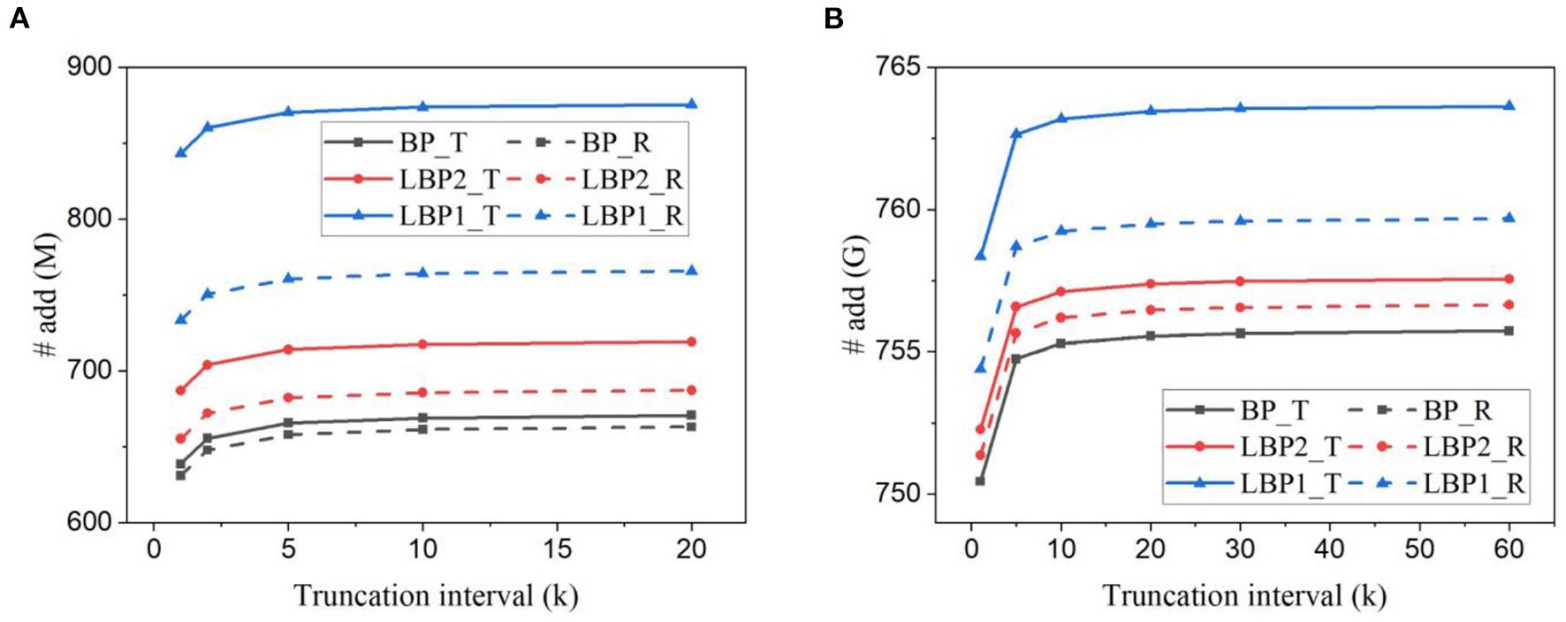
Number of additions estimated for training **(A)** LeNet-1 on EMNIST dataset and **(B)** LeNet-2 on DvsGesture dataset for one batch iteration. The batch size is 128.

**Figure 12:**
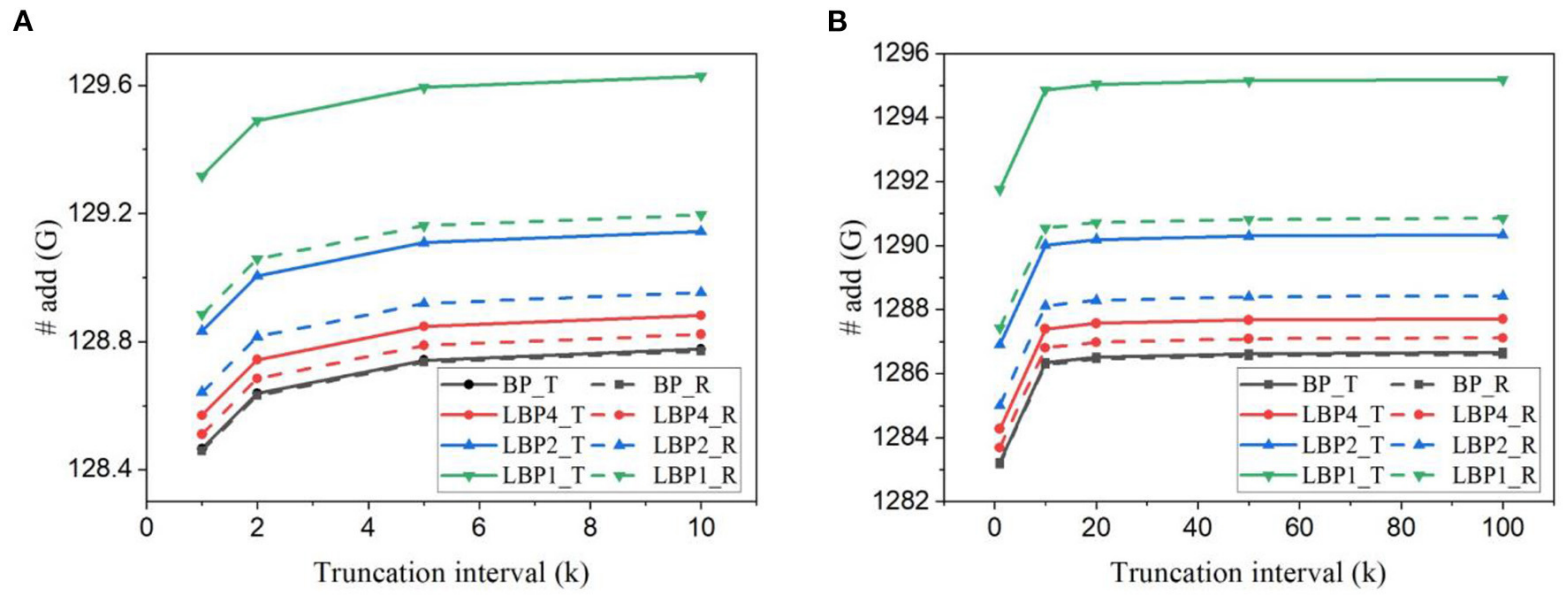
Number of additions estimated for training AlexNet **(A)** on CIFAR10 dataset and **(B)** on CIFAR10-DVS dataset.

Based on the analytical model above, we estimated the number of arithmetic operations involved in one batch training iteration, including additions and MACs. Figures 11A, B plot the results for LeNet trained on EMNIST dataset and DvsGesture dataset, respectively. Figures 12A, B show the results for AlexNet trained on CIFAR10 dataset and CIFAR10-DVS dataset, respectively. All the results reveal the same trend of change of accuracy affected by temporal truncation and local training. Temporal truncation does not lead to a notable reduction, less than 5%/0.7% in LeNet-1/2 and 0.3% reduction in AlexNet at maximum. However, LBPs cause more additions than BP, up to 32% in LeNet-1, only 1% in LeNet-2 and 0.66% in AlexNet. This large difference is due to the proportion of classifiers in the whole network. In small networks with local classifiers, such as LeNet-1, the classifier size is comparable to the size of the main network, which causes a large overhead. The use of random classifiers can reduce the overhead to 16% in LeNet-1. Thus, random classifiers are beneficial to small networks in this regard.

**Figure 13:**
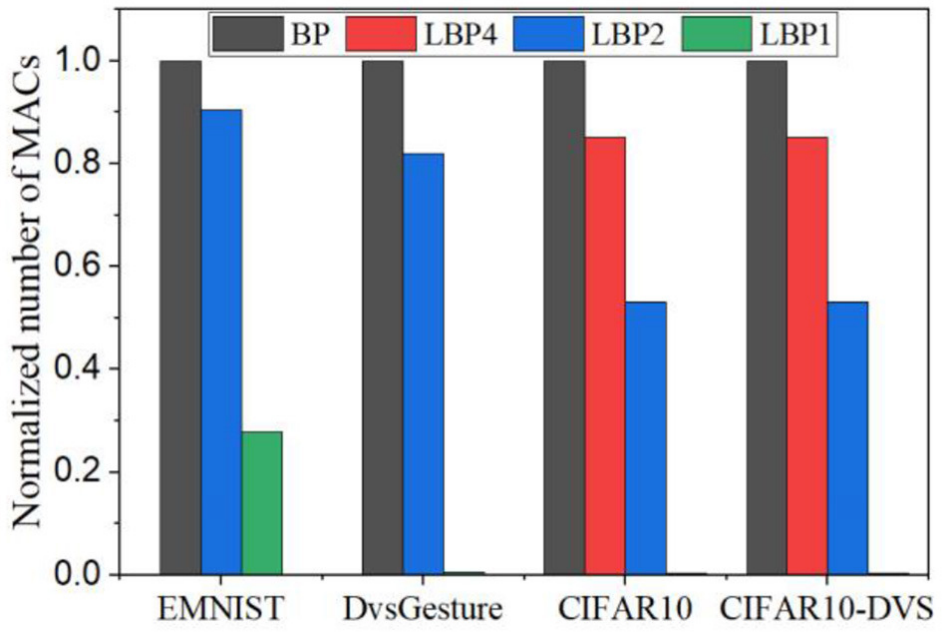
Normalized number of MACs estimated for training different networks averaged over training time windows for one batch iteration. For each task, the number of MACs was normalized over that required by BP.

The estimated number of MACs for different networks in one batch iteration is shown in Figure 13. We normalized the values over the number of MACs required by BP in each task. The number was also averaged over the training time window. The number of MACs is independent of truncation interval and the type of classifiers but dependent on the size of networks and local training blocks. Generally, multi-channel convolutional layers consume much more MACs than linear layers. In local training methods, convolutional operations between blocks are avoided because errors are not propagated. The local error propagation is from linear classifiers, leading to very small overhead. Increasing the number of local training blocks can significantly reduce MACs. Specifically, LBP1 leads to a 72% reduction in LeNet-1 and a 99% reduction in both LeNet-2 and AlexNet. The significant reduction in MACs is one of the most attractive benefits of local training methods, as it can greatly improve the training energy efficiency of SNNs and is not affected by BPTT.

## 5. Conclusion and discussions

We have investigated and analyzed the impact of the design variables on classification performance and computational cost in various tasks. In this section, we will address the important design problem with regard to the optimal choice of the variables while considering many performance aspects. The role of random classifiers will be discussed. Then, we will discuss the limitations of our training method and the promising solutions.

### 5.1. Summary

We have studied what roles the temporal truncation and local training play in affecting accuracy and computational cost including GPU memory cost, memory access, and arithmetic operations. The design space regarding the length of truncation interval and the size of local training blocks was explored. The impact of temporal truncation on accuracy depends on the type of datasets. It tends to decrease accuracy on frame-based datasets, while improves accuracy on DVS-recorded datasets with properly chosen intervals. Local training harms the classification performance when the size of network fits well with datasets, whereas it leads to improvement in the accuracy of the networks when overfitting is severe. In most cases, temporal truncation functions synergistically with local training. The combined effect helps slow down the decrease of accuracy and even improve accuracy in many cases. Both methods can contribute to a substantial reduction in GPU memory. Temporal truncation reduces memory access volume and has a negligible effect in lessening computational operations. Local training causes notable overhead in memory access and additions in small networks. However, it brings down the number of MACs remarkably.

### 5.2. How to determine the design variables?

**Figure 14:**
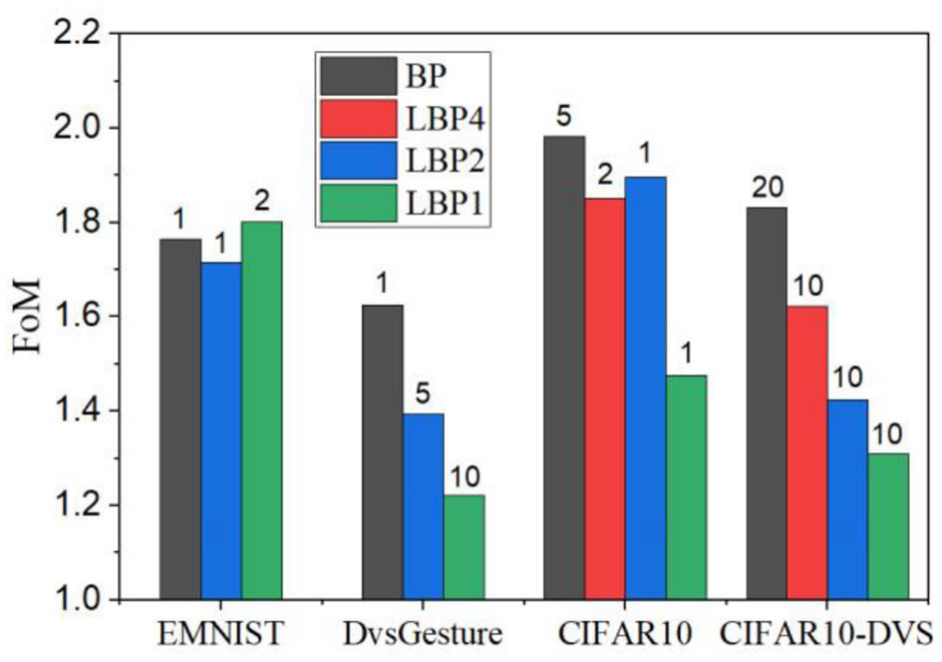
FoMs for different local training methods on different datasets. For each local training method, the smallest FoM is selected. On top of each bar, the value represents the truncation length.

It remains challenging in how to choose the degrees of temporal truncation and spatial locality, i.e., the values of (*k, n*). The choice depends on classification tasks and also the trade-off between classification performance and computational cost. For good classification performance, local training method could be promising with the block length larger than 1 and a good choice of k lies in the range from 2 to 10. For low computational cost, the best choice is undoubtedly the layer-wise local training with the truncation interval of 1. To provide a guidance for selecting (*k, n*), we define a figure of merit (FoM) considering both accuracy and computational cost equally as below


(18) $$\begin{array}{l} FoM=AL+0.25*\left(MC+\#MA+\#ADD+\#MAC\right) \end{array}$$

where *AL* is the accuracy loss, *MC* is the GPU memory cost, *#MA, #ADD*, and *#MAC* are the number of memory access, additions, and MAC, respectively. All the terms are normalized against the BPTT method. From the definition, a small FoM is desirable. Figure 14 displays the comparison among different local training methods across all the datasets under the defined FoM. For each local training method, the smallest FoM is selected, and the corresponding value of *k* is shown on top of each bar. From the comparison, the layer-wise local training method (LBP1) shows the best FoM on all the datasets except for EMNIST. In most cases, the best *k* lies in the range from 1 to 10. Specifically, the best values of (*k, n*) are (1, 2), (10, 1), (1, 1), and (10, 1) on EMNIST, DvsGesture, CIFAR10, and CIFAR10-DVS, respectively. It is worth noting that the proposed FoM considers the equal contribution from accuracy and computational cost and different definitions can be proposed to determine the design choice under practical application constraints.

**Figure 15:**
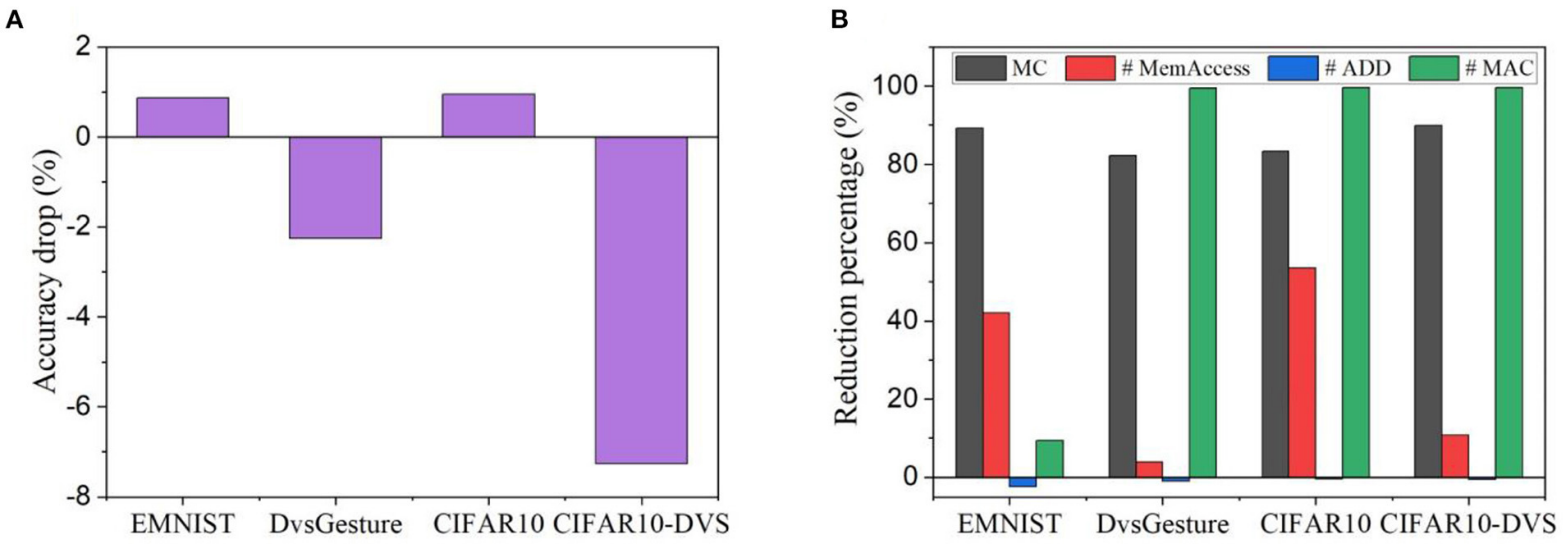
**(A)** Accuracy drop and **(B)** computational cost reduction of the selected method by the proposed FoM on all the datasets. The BPTT method is taken as the baseline for calculating the accuracy drop and computational cost reduction.

In Figures 15A, B, we summarized the accuracy drop and computational cost reduction of the best training design according to the proposed FoM. The BPTT method is taken as the baseline for calculating the accuracy drop and computational cost reduction. On EMNIST and CIFAR10, the accuracy drop is within 1%, whereas the accuracy can be improved by up to 7.26% in the other cases. On the other hand, the proposed training method leads to >80% reduction in GPU memory cost and >99% reduction in the number of MACs in most cases. On two datasets, the number of memory access is also considerably reduced by >40%. A negligible overhead in additions can be observed. Therefore, the proposed training method has been demonstrated to retain good classification performance or even improve it while achieving significant reduction in computational cost.

### 5.3. The role of random classifiers

The use of random classifiers in LBPs was proposed for its potential contribution in reducing computational cost in both ANNs and SNNs (Mostafa et al., 2018; Kaiser et al., 2020). No comparisons were made between random classifiers and trainable classifiers. Our work reveals a detailed comparison between them. Random classifiers cause worse accuracy when used with LBPs. Especially, the loss becomes more severe in LBP1. They are beneficial in reducing memory access and additions in small networks, but make negligible contributions to reduction in GPU memory, memory access, and additions in large networks. They have no effect on MACs. More specifically, in AlexNet trained on CIFAR10-DVS with the same (*k, n*), random classifiers can cause 20.16% accuracy drop with negligible improvement in computational cost compared to trainable classifiers. Therefore, our study shows that trainable classifiers have more considerable merit than random classifiers.

### 5.4. Limitations and future perspectives

The temporal truncation interval and local block length are two hyper-parameters that requires to be optimized in our proposed method. In our study, we have adopted a grid search to determine the optimal values. The results reflect that the optimal values vary from task to task and network to network, which poses a limitation on the applicability of the proposed method. A good choice of (*k, n*) has been discussed above under the proposed FoM. Although the optimal performance may not be achieved on all the tasks, it can still deliver promising improvement. Hyperparameter optimization has remained a challenge in deep learning. Most commonly in literatures, hyperparameters are chosen based on rules of thumb summarized in practice involving manual tuning. There exists many optimization algorithms. Classic approaches, such as random search (Bergstra and Bengio, 2012), Bayesian model (Snoek et al., 2012), and evolutional algorithms (Xiang and Zhining, 2019), are generally time consuming and may not converge. In recent years, gradient-descent based optimization methods have made it possible to directly optimize hyperparameters in the training loop, such as bilevel optimization (Franceschi et al., 2018). Thus, such optimization method could be a promising addition to our proposed training method to automate hyperparameter search for achieving optimal performance in various tasks.

TBPTT can be implemented in different ways. Instead of going through all the time steps in the backward pass during a truncation interval, the backward update can stop in the middle. In other words, the backward update can have a shorter time pass than the forward update. Cutting short the backward pass can furthermore reduce computational cost. Although in our work local training has been shown to improve classification performance in some cases, the intrinsic downside of local training method still remains and could considerably harm the performance of large-scale networks such as ResNets for more complex tasks such as ImageNet. As pointed out by Wang et al. (2021) local training is short-sighted and suffers from essential information loss while progressing along with the network. Many solutions were proposed to alleviate this issue. Wang et al. (2021) proposed an alternative loss function considering information preservation. Nokland and Eidnes applied an auxiliary loss function to create another backward pass for information flow (Nøkland and Eidnes, 2019). These proposals provide opportunities for further improvement in classification performance in our proposed training method.

## Data availability statement

The original contributions presented in the study are included in the article/supplementary material, further inquiries can be directed to the corresponding author.

## Author contributions

Conceptualization: WG and KS. Methodology: WG, MF, AE, and KS. Software, algorithms, and writing—original draft preparation: WG. Investigation and validation: WG and MF. Writing—review and editing: MF, AE, and KS. Supervision: AE and KS. Project administration: KS. All authors contributed to the article and approved the submitted version.

## References

[B1] Abderrahmane N. Lemaire E. Miramond B. (2020). Design space exploration of hardware spiking neurons for embedded artificial intelligence. Neur. Netw. 121, 366–386. 10.1016/j.neunet.2019.09.024 31593842

[B2] Aicher C. Foti N. J. Fox E. B. (2019). “Adaptively Truncating Backpropagation Through Time to Control Gradient Bias”, in *UAI 2019, the Conference on Uncertainty in Artificial Intelligence* (Tel Aviv-Yafo, Israel).

[B3] Amir A. Taba B. Berg D. Melano T. Mckinstry J. Nolfo C. D.. (2017). “A Low Power, Fully Event-Based Gesture Recognition System”, in *2017 IEEE Conference on Computer Vision and Pattern Recognition (CVPR)* 7388–7397. 10.1109/CVPR.2017.781

[B4] Amir A. Taba B. Berg D. J. Melano T. Mckinstry J. L. Nolfo C. D.. (2021). “Deep Residual Learning in Spiking Neural Networks”, in *Advances in Neural Information Processing Systems*.

[B5] Belilovsky E. Eickenberg M. Oyallon E. (2020). “Decoupled greedy learning of CNNs”, in *Proceedings of the 37th International Conference on Machine Learning*.

[B6] Bergstra J. Bengio Y. (2012). Random search for hyper-parameter optimization. J. Mach. Learn. Res. 13, 281–305.

[B7] Beyeler M. Oros N. Dutt N. Krichmar J. L. (2015). A GPU-accelerated cortical neural network model for visually guided robot navigation. Neur. Netw. 72, 75–87. 10.1016/j.neunet.2015.09.005 26494281

[B8] Bi G. -Q. Poo M. -M. (1998). Synaptic modifications in cultured hippocampal neurons: dependence on spike timing, synaptic strength, and postsynaptic cell type. J. Neurosci. 18, 10464–10472. 10.1523/JNEUROSCI.18-24-10464.1998 9852584 PMC6793365

[B9] Bohté S. M. Kok J. N. Poutré H. L. (2000). “SpikeProp: backpropagation for networks of spiking neurons”, in *ESANN 2000, European Symposiumon Artificial Neural Networks*.

[B10] Cohen G. Afshar S. Tapson J. C. Schaik A. V. (2017). “EMNIST: Extending MNIST to handwritten letters,” in 2017 International Joint Conference on Neural Networks (IJCNN) 2921–2926. 10.1109/IJCNN.2017.7966217

[B11] Davies M. Srinivasa N. Lin T. H. Chinya G. Cao Y. Choday S. H.. (2018). Loihi: A neuromorphic manycore processor with on-chip learning. IEEE Micro. 38, 82–99. 10.1109/MM.2018.112130359

[B12] Deng L. Wu Y. Hu X. Liang L. Ding Y. Li G.. (2020). Rethinking the performance comparison between SNNS and ANNS. Neur. Netw. 121, 294–307. 10.1016/j.neunet.2019.09.005 31586857

[B13] Diehl P. Cook M. (2015). Unsupervised learning of digit recognition using spike-timing-dependent plasticity. Front. Computat. Neurosci. 9, 99. 10.3389/fncom.2015.00099 26941637 PMC4522567

[B14] Diehl P. U. Neil D. Binas J. Cook M. Liu S. Pfeiffer M. (2015). “Fast-classifying, high-accuracy spiking deep networks through weight and threshold balancing”, in *2015 International Joint Conference on Neur. Netw. (IJCNN)* 1–8. 10.1109/IJCNN.2015.7280696

[B15] Ding J. Yu Z. Tian Y. Huang T. (2021). “Optimal ANN-SNN conversion for fast and accurate inference in deep spiking neural networks”, in *Proceedings of the Thirtieth International Joint Conference on Artificial Intelligence*. 10.24963/ijcai.2021/321

[B16] Esser S. K. Merolla P. A. Arthur J. V. Cassidy A. S. Appuswamy R. Andreopoulos A.. (2016). Convolutional networks for fast, energy-efficient neuromorphic computing. Proc. Nat. Acad. Sci. 113, 11441–11446. 10.1073/pnas.1604850113 27651489 PMC5068316

[B17] Fang W. Yu Z. Chen Y. Huang T. Masquelier T. Tian Y. (2021). Deep Residual Learning in Spiking Neural Networks. Adv. Neur. Inf. Proces. Syst. 34, 21056–21069.

[B18] Franceschi L. Frasconi P. Salzo S. Grazzi R. Pontil M. (2018). “Bilevel programming for hyperparameter optimization and meta-learning”, in *International Conference on Machine Learning* 1568–1577.

[B19] Furuya K. Ohkubo J. (2021). Semi-supervised learning combining backpropagation and STDP: STDP enhances learning by backpropagation with a small amount of labeled data in a spiking neural network. J. Phys. Soc. Japan 90, 074802. 10.7566/JPSJ.90.074802

[B20] Gao Y. Liu Y. Zhang H. Li Z. Zhu Y. Lin H.. (2020). “Estimating GPU memory consumption of deep learning models”, in *Proceedings of the 28th ACM Joint Meeting on European Software Engineering Conference and Symposium on the Foundations of Software Engineering* (Virtual Event, USA: Association for Computing Machinery). 10.1145/3368089.3417050

[B21] Guo W. Yantir H. E. Fouda M. E. Eltawil A. M. Salama K. N. (2021). “Toward the optimal design and FPGA implementation of spiking neural networks,” in IEEE Transactions on Neural Networks and Learning Systems 1–15. 33571097 10.1109/TNNLS.2021.3055421

[B22] Gütig R. Sompolinsky H. (2006). The tempotron: a neuron that learns spike timing–based decisions. Nature Neurosci. 9, 420–428. 10.1038/nn1643 16474393

[B23] Han S. Pool J. Tran J. Dally W. J. (2015). “Learning both weights and connections for efficient neural networks”, in *Proceedings of the 28th International Conference on Neural Information Processing Systems - Volume 1*. (Montreal, Canada: MIT Press).

[B24] He W. Wu Y. Deng L. Li G. Wang H. Tian Y.. (2020). Comparing SNNs and RNNs on neuromorphic vision datasets: Similarities and differences. Neur. Netw. 132, 108–120. 10.1016/j.neunet.2020.08.001 32866745

[B25] Höppner S. Yan Y. Dixius A. Scholze S. Partzsch J. Stolba M.. (2021). The SpiNNaker 2 processing element architecture for hybrid digital neuromorphic computing. ArXiv abs/2103.08392. 10.48550/arXiv.2103.08392

[B26] Jin Y. Zhang W. Li P. (2018). “Hybrid macro/micro level backpropagation for training deep spiking neural networks”, in *Proceedings of the 32nd International Conference on Neural Information Processing Systems*. (Montréal, Canada: Curran Associates Inc.).

[B27] Kaiser J. Mostafa H. Neftci E. (2020). Synaptic Plasticity Dynamics for Deep Continuous Local Learning (DECOLLE). Front. Neurosci. 14, 424. 10.3389/fnins.2020.00424 32477050 PMC7235446

[B28] Karen Simonyan A. Z. (2014). “Very deep convolutional networks for large-scale image recognition”, in *The International Conference on Learning Representations (ICLR)*.

[B29] Kheradpisheh S. R. Ganjtabesh M. Thorpe S. J. Masquelier T. (2018). STDP-based spiking deep convolutional *Neur. Netw*. for object recognition. Neur. Netw. 99, 56–67. 10.1016/j.neunet.2017.12.005 29328958

[B30] Kheradpisheh S. R. Masquelier T. (2020). Temporal backpropagation for spiking neural networks with one spike per neuron. Int. J. Neural Syst. 30, 2050027. 10.1142/S0129065720500276 32466691

[B31] Kim S. Park S. Na B. Yoon S. (2020). Spiking-YOLO: Spiking neural network for energy-efficient object detection. Proc. AAAI Conf. Artif. Intell. 34, 11270–11277. 10.1609/aaai.v34i07.6787

[B32] Kim Y. Panda P. (2021). Optimizing deeper spiking neur. netw. for dynamic vision sensing. Neur. Netw. 144, 686–698. 10.1016/j.neunet.2021.09.022 34662827

[B33] Kingma D. P. Ba J. (2015). “Adam: A Method for Stochastic Optimization”, in *ICLR 2015, the International Conference on Learning Representations*.

[B34] Krizhevsky A. (2009). Learning multiple layers of features from tiny images. Available online at: http://www.cs.utoronto.ca/~kriz/learning-features-2009-TR.pdf (accessed December 8, 2021).

[B35] Krizhevsky A. Sutskever I. Hinton G.E. (2017). ImageNet classification with deep convolutional neural networks. Commun. ACM 60, 84–90. 10.1145/3065386

[B36] Lecun Y. Bottou L. Bengio Y. Haffner P. (1998). Gradient-based learning applied to document recognition. Proc. IEEE 86, 2278–2324. 10.1109/5.726791

[B37] Lee C. Panda P. Srinivasan G. Roy K. (2018). Training deep spiking convolutional neural networks with STDP-based unsupervised pre-training followed by supervised fine-tuning. Front. Neurosci. 12, 435. 10.3389/fnins.2018.00435 30123103 PMC6085488

[B38] Lee C. Sarwar S. S. Panda P. Srinivasan G. Roy K. (2020). Enabling spike-based backpropagation for training deep neural network architectures. Front. Neurosci. 14, 119. 10.3389/fnins.2020.00119 32180697 PMC7059737

[B39] Lee J. H. Delbruck T. Pfeiffer M. (2016). Training Deep Spiking neural networks Using Backpropagation. Front. Neurosci. 10, 1–13. 10.3389/fnins.2016.00508 27877107 PMC5099523

[B40] Li G. Müller M. Ghanem B. Koltun V. (2021). “Training graph neural networks with 1000 layers”, in *International Conference on Machine Learning* 6437–6449.

[B41] Li H. Liu H. Ji X. Li G. Shi L. (2017). CIFAR10-DVS: An event-stream dataset for object classification. Front. Neurosci. 11, 309. 10.3389/fnins.2017.00309 28611582 PMC5447775

[B42] Li Z. Liu F. Yang W. Peng S. Zhou J. (2021). “A survey of convolutional neural networks: analysis, applications, and prospects,” in IEEE Transactions on Neural Networks and Learning Systems 1–21. 34111009 10.1109/TNNLS.2021.3084827

[B43] Lin Y. Ding W. Qiang S. Deng L. Li G. (2021). ES-ImageNet: A million event-stream classification dataset for spiking neural networks. Front. Neurosci. 15, 726582. 10.3389/fnins.2021.726582 34899154 PMC8655353

[B44] Liu F. Zhao W. Chen Y. Wang Z. Jiang L. (2022). “SpikeConverter: An efficient conversion framework zipping the gap between artificial neural networks and spiking neural networks,” in Proceedings of the AAAI Conference on Artificial Intelligence 36, 1692–1701. 10.1609/aaai.v36i2.20061

[B45] Liu F. Zhao W. Chen Y. Wang Z. Yang T. Jiang L. (2021). SSTDP: Supervised spike timing dependent plasticity for efficient spiking neural network training. Front. Neurosci. 15, 756876. 10.3389/fnins.2021.756876 34803591 PMC8603828

[B46] Ma C. Xu J. Yu Q. (2021). “Temporal dependent local learning for deep spiking neural networks”, in *2021 International Joint Conference on Neural Networks (IJCNN)* 1–7. 10.1109/IJCNN52387.2021.9534390

[B47] Mahmud M. Kaiser M. S. Hussain A. Vassanelli S. (2018). Applications of deep learning and reinforcement learning to biological data. IEEE Trans. Neur. Netw. Learn. Syst. 29, 2063–2079. 10.1109/TNNLS.2018.2790388 29771663

[B48] Marquez E. S. Hare J. S. Niranjan M. (2018). Deep cascade learning. IEEE Trans. Neur. Netw. Learn. Syst. 29, 5475–5485. 10.1109/TNNLS.2018.2805098 29993618

[B49] Meng Q. Yan S. Xiao M. Wang Y. Lin Z. Luo Z. -Q. (2022). Training much deeper spiking *Neur. Netw*. with a small number of time-steps. Neur. Netw. 153, 254–268. 10.1016/j.neunet.2022.06.001 35759953

[B50] Mirsadeghi M. Shalchian M. Kheradpisheh S. R. Masquelier T. (2021). STiDi-BP: Spike time displacement based error backpropagation in multilayer spiking neural networks. Neurocomputing 427, 131–140. 10.1016/j.neucom.2020.11.052

[B51] Mostafa H. Ramesh V. Cauwenberghs G. (2018). Deep supervised learning using local errors. Front. Neurosci. 12, 608. 10.3389/fnins.2018.00608 30233295 PMC6127296

[B52] Naveros F. Luque N. R. Ros E. Arleo A. (2020). VOR adaptation on a humanoid iCub robot using a spiking cerebellar model. IEEE Trans. Cybern. 50, 4744–4757. 10.1109/TCYB.2019.2899246 30835236

[B53] Nøkland A. Eidnes L. H. (2019). “Training neural networks with local error signals”, in *International Conference on Machine Learning* 4839–4850.

[B54] Orchard G. Jayawant A. Cohen G. K. Thakor N. (2015). Converting static image datasets to spiking neuromorphic datasets using saccades. Front. Neurosci. 9, 437. 10.3389/fnins.2015.00437 26635513 PMC4644806

[B55] Park S. Yoon S. (2021). Training energy-efficient deep spiking neural networks with time-to-first-spike coding. ArXiv abs/2106.02568. 10.48550/arXiv.2106.02568

[B56] Pascanu R. Mikolov T. Bengio Y. (2013). “On the difficulty of training recurrent neural networks”, in *Proceedings of the 30th International Conference on Machine Learning* (Atlanta, GA, USA: JMLR.org).

[B57] Paszke A. (2019). “PyTorch: An imperative style, high-performance deep learning library,” in Advances in Neural Information Processing Systems 8024–8035.

[B58] Sengupta A. Ye Y. Wang R. Liu C. Roy K. (2019). Going deeper in spiking neural networks: VGG and residual architectures. Front. Neurosci. 13, 95. 10.3389/fnins.2019.00095 30899212 PMC6416793

[B59] Shrestha S. Orchard G. (2018). “SLAYER: Spike Layer Error Reassignment in Time”, in *Advances in Neural Information Processing Systems* 31

[B60] Snoek J. Larochelle H. Adams R. P. (2012). “Practical Bayesian optimization of machine learning algorithms”, in *Proceedings of the 25th International Conference on Neural Information Processing Systems - Volume 2*. (Lake Tahoe, Nevada: Curran Associates Inc.).

[B61] Srinivasan G. Panda P. Roy K. (2018). STDP-based unsupervised feature learning using convolution-over-time in spiking neural networks for energy-efficient neuromorphic computing. J. Emerg. Technol. Comput. Syst. 14, 44. 10.1145/3266229

[B62] Srivastava N. Hinton G. Krizhevsky A. Sutskever I. Salakhutdinov R. (2014). Dropout: a simple way to prevent neural networks from overfitting. J. Mach. Learn. Res. 15, 1929–1958.33259321

[B63] Sutskever I. (2013). Training Recurrent Neural Networks. Toronto, ON, Canada: University of Toronto.

[B64] Sutskever I. Martens J. Dahl G. Hinton G. (2013). “On the importance of initialization and momentum in deep learning”, in *Proceedings of the 30th International Conference on Machine Learning*.

[B65] Tavanaei A. Ghodrati M. Kheradpisheh S. R. Masquelier T. Maida A. (2019). Deep learning in spiking neural networks. Neur. Netw. 111, 47–63. 10.1016/j.neunet.2018.12.002 30682710

[B66] Tavanaei A. Maida A. (2019). BP-STDP: Approximating backpropagation using spike timing dependent plasticity. Neurocomputing 330, 39–47. 10.1016/j.neucom.2018.11.01428095200

[B67] Wang Y. Ni Z. Song S. Yang L. Huang G. (2021). “Revisiting Locally Supervised Learning: an Alternative to End-to-end Training”, in *ICLR 2021, the International Conference on Learning Representations*.

[B68] Williams R. J. Zipser D. (1995). “Gradient-based learning algorithms for recurrent networks and their computational complexity,” in Backpropagation: Theory, Architectures, and Applications (New York, NY: L. Erlbaum Associates Inc.) 433–486.

[B69] Wu J. Chua Y. Zhang M. Li G. Li H. Tan K. C. (2021). “A tandem learning rule for effective training and rapid inference of deep spiking neural networks,” in IEEE Transactions on Neural Networks and Learning Systems 1–15. 34288879 10.1109/TNNLS.2021.3095724

[B70] Wu Y. Deng L. Li G. Zhu J. Shi L. (2018). Spatio-temporal backpropagation for training high-performance spiking neural networks. Front. Neurosci. 12, 331. 10.3389/fnins.2018.00331 29875621 PMC5974215

[B71] Wu Y. Deng L. Li G. Zhu J. Shi L. (2019). Direct training for spiking neural networks: faster, larger, better. Proc. AAAI Conf. Artif. Intell. 33, 1311–1318. 10.1609/aaai.v33i01.33011311

[B72] Xiang W. Zhining Y. (2019). “Neural network hyperparameter tuning based on improved genetic algorithm”, in *Proceedings of the 2019 8th International Conference on Computing and Pattern Recognition* (Beijing, China: Association for Computing Machinery). 10.1145/3373509.3373554

[B73] Young T. Hazarika D. Poria S. Cambria E. (2018). Recent trends in deep learning based natural language processing. IEEE Comput. Intell. Magaz. 13, 55–75. 10.1109/MCI.2018.284073834324453

[B74] Yu Q. Song S. Ma C. Wei J. Chen S. Tan K. C. (2021). “Temporal encoding and multispike learning framework for efficient recognition of visual patterns,” in IEEE Transactions on Neural Networks and Learning Systems 1–13. 33531306 10.1109/TNNLS.2021.3052804

[B75] Zhao Z. Zheng P. Xu S. Wu X. (2019). “Object detection with deep learning: a review,” in IEEE Transactions on Neural Networks Learning Systems 30, 3212–3232. 10.1109/TNNLS.2018.2876865 30703038

[B76] Zheng H. Wu Y. Deng L. Hu Y. Li G. (2021). “Going deeper with directly-trained larger spiking neural networks”, in *AAAI-21, The Thirty-Fifth AAAI Conference on Artificial Intelligence*. 10.1609/aaai.v35i12.17320

